# QSAR, ADMET, molecular docking, and dynamics studies of 1,2,4-triazine-3(2H)-one derivatives as tubulin inhibitors for breast cancer therapy

**DOI:** 10.1038/s41598-024-66877-2

**Published:** 2024-07-16

**Authors:** Mohamed Moussaoui, Soukayna Baammi, Hatim Soufi, Mouna Baassi, Achraf El Allali, M. E. Belghiti, Rachid Daoud, Said Belaaouad

**Affiliations:** 1 Laboratory of Physical Chemistry of Materials, Faculty of Sciences Ben M’Sick, Hassan II University of Casablanca, Casablanca, Morocco; 2 Bioinformatics Laboratory, College of Computing, Mohammed VI Polytechnic University, Ben Guerir, Morocco; 3 Laboratory of Nernest Technology, 163 Willington Street, Sherbrook, QC J1H5C7 Canada; 4 Chemical and Biochemical Sciences-Green Processing Engineering, Mohammed VI Polytechnic University, Ben Guerir, Morocco

**Keywords:** QSAR, Molecular docking, ADMET, 1,2,4-triazin-3(2H)-one, Breast cancer, Anticancer, Biochemistry, Cancer, Drug discovery, Chemistry

## Abstract

Breast cancer remains a leading cause of cancer-related deaths among women globally, necessitating the development of more effective therapeutic agents with minimal side effects. This study explores novel 1,2,4-triazine-3(2H)-one derivatives as potential inhibitors of Tubulin, a pivotal protein in cancer cell division, highlighting a targeted approach in cancer therapy. Using an integrated computational approach, we combined quantitative structure–activity relationship (QSAR) modeling, ADMET profiling, molecular docking, and molecular dynamics simulations to evaluate and predict the efficacy and stability of these compounds. Our QSAR models, developed through rigorous statistical analysis, revealed that descriptors such as absolute electronegativity and water solubility significantly influence inhibitory activity, achieving a predictive accuracy (R^2^) of 0.849. Molecular docking studies identified compounds with high binding affinities, particularly Pred28, which exhibited the best docking score of − 9.6 kcal/mol. Molecular dynamics simulations conducted over 100 ns provided further insights into the stability of these interactions. Pred28 demonstrated notable stability, with the lowest root mean square deviation (RMSD) of 0.29 nm and root mean square fluctuation (RMSF) values indicative of a tightly bound conformation to Tubulin. The novelty of this work lies in its methodological rigor and the integration of multiple advanced computational techniques to pinpoint compounds with promising therapeutic potential. Our findings advance the current understanding of Tubulin inhibitors and open avenues for the synthesis and experimental validation of these compounds, aiming to offer new solutions for breast cancer treatment.

## Introduction

Breast cancer remains the most diagnosed cancer among women globally. According to the World Health Organization's 2023 report^1^, each year, more than 2.3 million women are diagnosed with breast cancer, making it the most common cancer in the world affecting all adults. This accounts for nearly 25% of all cancers in women. These statistics highlight the persistent challenge breast cancer poses and underscore the critical need for developing more effective therapeutic strategies.

The 1,2,4-triazine-3(2H)-one derivatives have emerged as a promising class of anticancer agents, with several studies reporting their potent anticancer activity against^2^ breast cancer cell lines. These derivatives act by disrupting microtubule dynamics, which are essential for cell division^3^, through their interaction with Tubulin, specifically the Colchicine binding site. The Tubulin protein was chosen as the focal point of our research due to its critical role in regulating microtubule dynamics and its active involvement in the division of breast cancer cells. Our decision to focus on Tubulin-Colchicine as the target protein for our study was made after thoughtful deliberation regarding its importance in cancer treatment. Tubulin proteins are key components of the cellular cytoskeleton and are crucial for cell division, making them an attractive target for cancer therapy. Colchicine-binding to Tubulin disrupts its polymerization, thereby inhibiting mitosis and cell proliferation. Given the mechanism of action and the success of Colchicine and its analogs in cancer treatment, targeting the Tubulin-Colchicine site provides a rational approach for novel anticancer drug design. Furthermore, the 1,2,4-triazine-3(2H)-one derivatives were chosen for their structural similarities to known Tubulin-Colchicine inhibitors, as well as their promising pharmacokinetic profiles, making them viable candidates for further investigation^4–7^.

It is important to note that the focus of this study is on targeting the Tubulin-Colchicine binding site as a general approach to breast cancer therapy. While Tubulin inhibitors have shown efficacy in various types of breast cancer, the applicability of our 1,2,4-triazine-3(2H)-one derivatives to specific subtypes such as Triple-Negative Breast Cancer (TNBC) has not been investigated in this study. Future work should delve into the subtype-specific efficacy of these compounds.

In this context, computational tools such as quantitative structure–activity relationship (QSAR) modeling, molecular docking, and molecular dynamics simulations are pivotal in enhancing the efficiency and efficacy of drug discovery processes. QSAR modeling is crucial for understanding the relationship between chemical structures and their biological activities. This method allows researchers to predict the activity of new compounds based on their chemical properties, thereby streamlining the identification of promising candidates for further experimental validation. QSAR models provide insights into the essential features influencing drug efficacy and safety, guiding the synthesis of compounds with optimized pharmacological profiles^8,9^.

Molecular docking techniques simulate the interaction between a molecule and its biological target, typically a protein involved in disease pathways. By predicting how small molecules fit into the binding sites of target proteins, molecular docking helps to ascertain the binding affinity and interaction mechanisms, crucial for assessing the therapeutic potential of new drugs. This approach is instrumental in refining the molecular structures of drug candidates to enhance their specificity and potency against cancer targets^10,11^.

Molecular dynamics simulations further extend our understanding of drug-target interactions over time, offering insights into the stability and behavior of these interactions under physiologically relevant conditions. By examining the dynamics of protein–ligand complexes through simulations, we can assess the flexibility and conformation changes of both the target and the ligands. This method provides valuable data on the durability of the binding over time, essential for predicting the in vivo efficacy and stability of therapeutic candidates.

In our study, we employ these advanced computational techniques to explore the potential of 1,2,4-triazine-3(2H)-one derivatives as inhibitors of Tubulin, a key protein in cell division and a validated target in cancer treatment. Integrating QSAR modeling, ADMET^12^, molecular docking, and molecular dynamics simulations allows us to comprehensively predict and analyze the interactions, stability, and efficacy of these compounds. This holistic approach not only informs the drug design process but also aligns with the broader goal of accelerating the discovery and optimization of effective cancer therapies.

## Materials and methods

### Data set

Based on the research of Eissa et al. a database of 32 1,2,4-triazine-3 (2H)-one derivative with their inhibitory efficacy against the MCF-7 breast cancer cell line was created^13^, has been compiled to perform this study. The selection of this database is due to the following reasons: (i) the compounds in our database represent structural diversity with pIC50 (3.460–4.963), (ii) actually, the 1,2,4-triazin-3(2H)-one derivatives present a privileged heterocyclic for drug design in therapeutic medicinal. The reported IC50 values of compounds have been converted into corresponding pIC50 (−log IC50) (Table S1).

To ensure the robustness and reliability of our QSAR model, we adopted an 80:20 ratio for dividing our dataset into training and test sets. This ratio is widely recognized in computational drug design for balancing extensive model training with adequate external validation. We implemented a randomized split to avoid potential biases related to the order of data entries, ensuring that both subsets are representative of the overall dataset. This approach allows for comprehensive training of the model on diverse chemical entities while providing a substantial and unbiased test set for evaluating the model's predictive accuracy on unseen data.

### Molecular descriptors

Currently, several different in QSAR research, molecules are employed as descriptors. The results may be used to forecast the activity of substances that have not been tested after being validated. The Gaussian 09W program was used to compute the electronic descriptors^14^. The 32 1,2,4-triazine-3(2H)-one derivatives' geometries were optimized using the DFT approach and the B3LYP functional^15^ and 6-31G (p, d) base set^16–18^. Then, several related structural parameters were selected from the results of the quantum computation as follows: highest occupied molecular orbital energy (E_HOMO_), lowest unoccupied molecular orbital energy (E_LUMO_), dipole moment (μm), total energy (TE), absolute hardness (η), absolute electronegativity (χ) and reactivity index (ω)^19^. The η, χ and ω were determined using the following equations:

$$\upeta = \frac{{\text{E}}_{\text{LUMO}}-{\text{E}}_{\text{HOMO}}}{2},\upchi = \frac{{\text{E}}_{\text{LUMO}}+ {\text{E}}_{\text{HOMO}}}{2},\upomega = \frac{{\upmu }^{2}}{2\upeta }$$

ChemOffice software 16.0^20^ was used to calculate the topological descriptors, as follows: molecular weight (MW), Number of HBond Acceptors (NHA), Number of HBond Donors (NHD), Octanol–Water Partition Coefficient (LogP), Water Solubility (LogS), Balaban Index (J), Molecular Topological Index (MTI), Polar Surface Area (PSA), Radius (R_DWV_), Shape Coefficient (I), Sum of Valence Degrees (SVD), Wiener Index (WI) and Number Rotatable Bonds (N_ROT_).

The selection of molecular descriptors was a critical step in the development of our QSAR model. We employed a combination of statistical analyses and biological reasoning to arrive at our final set of descriptors. Each descriptor was chosen based on its ability to provide unique and non-redundant information about the biological activity of the compounds under study. For instance, the descriptor 'χ' (absolute electronegativity) was selected due to its relevance in predicting molecular interactions, while 'LogS' was chosen for its importance in solubility prediction. Furthermore, the selected descriptors showed low multicollinearity and were statistically significant in the model according to the variance inflation factor (VIF) and p-values.

### Statistical analysis

Developing empirical models that connect a compound's biological activity to its chemical make-up is the goal of a quantitative structure–activity relationship (QSAR) research. In this QSAR study, the chemical structure is described quantitatively and the link between the chemical structure and biological activity is described mathematically. These 24 descriptors are produced for the 32 compounds using the Gaussian 09W and ChemOffice programmers to illustrate the structure–activity link.

Statistical techniques based on principal component analysis (PCA) are used to examine the quantitative characteristics of the substituted 1,2,4-triazine-3(2H)-one^21^ using XLSTAT version 2019 software^22^. PCA is a helpful statistical method for collecting all of the data included in the compound structures. Understanding the distribution of the chemicals is also made easier by it^23^. This statistical approach is mostly descriptive and seeks to visualize the most relevant information from the data, as shown in Tables S1 and S2.

The structure–activity link is modeled employing a descendent selection and variable removal multiple linear regression (MLR) approach. It is a mathematical technique designed to lessen the difference between observed and predicted values.

XLSTAT version 2019 was used to create the MLR model. Equations are supported by the correlation coefficient (R), mean squared error (MSE), Fisher's criteria (F), and significance level (P) to determine the pIC50.

1 $${R}^{2}=1-\frac{\sum {({Y}_{Obs}-{Y}_{pred})}^{2}}{\sum {({Y}_{pred}-{\overline{Y} }_{Obs})}^{2}}$$

2 $${R}_{test}^{2}=1-\frac{\sum {({Y}_{pred(test)}-{Y}_{Obs(test)})}^{2}}{\sum {({Y}_{Obs(test)}-{\overline{Y} }_{pred(train)})}^{2}}$$

3 $${Q}_{CV}^{2}=1-\frac{\sum {({Y}_{Obs}-{Y^{\prime}}_{pred})}^{2}}{\sum {({Y^{\prime}}_{pred}-{\overline{Y} }_{Obs})}^{2}}$$

4 $$MSE=\frac{\sum ({Y}_{Obs}-{Y}_{pred})}{N}$$

5 $${F}_{test}=\frac{\sum {({Y}_{pred}-\overline{{Y }_{pred}})}^{2}}{\sum {({Y}_{Obs}-{Y}_{pred})}^{2}}*\frac{N-p-1}{p}$$

where ${\text{Y}}_{\text{obs}}$ is the value of the observed response, ${\text{Y}}_{\text{pred}}$ is the value of the predicted response, ${\overline{\text{Y}} }_{\text{pred}}$ is the average value of observed/predicted responses, and p is the number of explicative variables in the model, and n is the number of individuals.

The final QSAR models were validated internally and externally. The internal validation procedure used was leave-one-out (LOO) cross-validation. The predicted values for the internal validation set were gathered, and the accuracy of the prediction of the pIC50 was evaluated using cross-validated R^2^ (Q^2^-LOO).

Additionally, the Golbraikh and Tropsha-proposed parameters for calculating a QSAR model's external predictability were derived^24^. In light of this, a QSAR model is deemed predictive if the following criteria are met:

- R^2^ > 0.6
- Q^2^ > 0.5
- $$\frac{{\text{R}}^{2}-{\text{R}}_{0}^{2}}{{\text{R}}^{2}}<0.1 \;\; \text{ and }\;\; 0.85 \le \text{k}\le 1.15 \;\; \text{ or} \;\; \frac{{\text{R}}^{2}-{{\text{R}}^{{{\prime}}}}_{0}^{2}}{{\text{R}}^{2}}<0.1\text{ and }0.85 \le \text{k}^{\prime}\le 1.15$$
- $$\left|{R}_{0}^{2}-\left.{R^{\prime}}_{0}^{2}\right|<0.3\right.$$

R^2^ and ${\text{R}}_{\text{0}}^{2}$ re the square correlation coefficients between the observed and the predicted activity values with and without intercept, respectively, while ${\text{R}}_{0}^{{\prime}2}$ represents the same information as ${\text{R}}_{0}^{2}$ does but with inverted axis (linear regression between the predicted against the observed values). These parameters can be calculated as follows:

6 $${\text{R}}_{0}^{2}=1-\frac{\sum {({\text{Y}}_{\text{pred}}-{\text{Y}}_{\text{obs}}^{{\prime}0})}^{2}}{\sum {({\text{Y}}_{\text{pred}}-{\overline{\text{Y}} }_{\text{pred}})}^{2}}$$

7 $${\text{R}}_{0}^{{\prime}2}=1-\frac{\sum {({\text{Y}}_{\text{obs}}-{\text{Y}}_{\text{pred}}^{{\prime}0})}^{2}}{\sum {({\text{Y}}_{\text{obs}}-{\overline{\text{Y}} }_{\text{obs}})}^{2}}$$

where ${\overline{\text{Y}} }_{\text{pred}}$ and ${\overline{\text{Y}} }_{\text{obs}}$ refer to the mean values of the predicted and observed activity data, respectively. The regression lines through the origin are defined by ${Y^{\prime}}_{obs}^{0}=k {Y}_{pred}$ and ${Y^{\prime}}_{pred}^{0}={k}^{\prime} {Y}_{obs}$, while the slopes k and k’ are calculated as follows:

8 $$k=\frac{\sum {Y}_{obs}{Y}_{pred}}{\sum {Y}_{pred}^{2}}$$

9 $$k^{\prime}=\frac{\sum {Y}_{obs}{Y}_{pred}}{\sum {Y}_{obs}^{2}}$$

Likewise, ${R}_{m}^{2}$ metrics proposed by Ojha et al.^25^ for external validation were calculated to further evaluate the correlation between the observed and predicted activity:

10 $${\overline{R} }_{m}^{2}= \frac{\left({R}_{m}^{2}+{R^{\prime}}_{m}^{2}\right)}{2}$$

11 $${\Delta R}_{m}^{2}= \left|{R}_{m}^{2}-\left.{R^{\prime}}_{m}^{2}\right|\right.$$

where

12 $${R}_{m}^{2}= {R}^{2}\times \left(1-\sqrt{\left({R}^{2}-{R}_{0}^{2}\right)}\right)$$

and

13 $${R^{\prime}}_{m}^{2}= {R}^{2}\times \left(1-\sqrt{\left({R}^{2}-{R^{\prime}}_{0}^{2}\right)}\right)$$

#### Y-Randomization test

Randomization of the response variable and the Y-randomization test were employed to determine the model's robustness. The whole training set's calculations are redone for this test using randomly generated activities.

The Q^2^ and R^2^ of the new QSAR models were lower than those of the original models. To rule out the potential of random correlation, this approach was used. Higher Q^2^ and R^2^ values indicate that structural redundancy and random correlation prevent the generation of a suitable QSAR for this dataset^26^.

The Y-randomization test also calculates the coefficient of determination, or cR^2^_p_ value, which is stated to need to be larger than 0.5 to pass the test^27^:

14 $${cR}_{p}^{2}=R*\sqrt{({R}^{2}-{(Average {R}_{Rand})}^{2}}$$

#### Model applicability domain

A model cannot be used to predict the biological activity for the entire chemicals in the universe except for those in its region of reliable/acceptable prediction, which is defined in terms of descriptors contained in the model. This region is known as the applicability domain (AD) of the model. In this study, the AD of the developed model was defined using the extent of the extrapolation method. This method employs leverage h values of dataset molecules and the standardized prediction residual (SDR) of the models to define their AD. The result of this method is often visualized by the plot of h versus SDR (Williams plot)^28^.

Leverage h is a special type of distance measures used to show similarity/dissimilarity among objects and it’s obtained as the diagonal element of a hat matrix hi:

15 $${h}_{i}={x}_{i}^{T}({X}^{T}X{)}^{-1}{x}_{i}$$

where each of the compounds as defined by i can take values from 1, 2,…, n.

Within the model that was developed, xi is taken as the row-vector descriptor for the main query complex. Additionally, X is the result of $n\times (k-1)$ matrix of k model and the descriptor values for the n training set compounds. In addition, T (depicted as a superscript number), refers to the transpose of matrix/vector^29^.

In general, the AD of the models in the research was defined as a square region with vertical boundary 0 < hi < h* and horizontal boundary −3 < SDR < 3, where hi’s were molecules leverages values and h* was the models warning leverage expressed as:

16 $${h}^{*}=3\times \frac{k+1}{n}$$

In Eq. (16)^30^, k is the number of descriptors in the model, and n is the number of training set molecules. Standardized residual (SDR) was calculated with the equation below:

17 $$SDR= \frac{{Y}_{obs}-{Y}_{pred}}{\sqrt{\frac{{\sum }_{i=1}^{n}{\left({Y}_{obs}-{Y}_{pred}\right)}^{2}}{n}}}$$

In Eq. (17), ${Y}_{obs}$ and ${Y}_{pred}$ are observed and predicted response respectively for either training or test set molecules, and n is the number of dataset molecules.

### Molecular docking studies

The crystal structure of human Tubulin-Colchicine was retrieved from the Protein Data Bank (PDB code: 1SA0, resolution 3.58 Å)^31^ and was used for the MD study. The protein preparation was further conducted by AutoDock Tools^32,33^: All ligands were extracted from the protein structure, missing residues were added and polar hydrogen atoms, Kollman charges and applying Gasteiger Marsili charges were added and removing water molecules were added using AutoDock Tools. For structures that were modelled using a template, energy minimization was carried out using using ChemOffice program with Minimum RMS Gradient of 0.010 to relieve any steric clashes and to optimize the geometry. The energy-minimized structure was then used for subsequent docking studies. The grid maps were created at 40 $\text{\AA }$ in all three dimensions (X, Y, Z), the center of this box is determined by the coordinates X = 117.219, Y = 90.179, and Z = 6.289 angstrom (Å) with a default grid space size of 0.375 $\text{\AA }$. 2D and 3D ligand interactions were visualized using Discovery studio to evaluate the strength of ligand–protein interactions.

### Molecular dynamic studies

To explore the structure–function relationship, molecular dynamics simulations (MD) were conducted with GROMACS 2019.3^34^ on the docked complexes comprising compounds Pred3, Pred13, Pred28, and Colchicine. The protein utilized the CHARMM27 force field^35^, while ligand topologies were developed via the Swissparam server^36^. Each complex was placed in a dodecahedral box with a 1.0 nm edge length, filled with TIP3P water, and neutralized with Na + ions^37^. Energy minimization was achieved using the steepest descent method, targeting a maximum force of 1000 kJ/mol/nm^38^. Two 100 ps simulations were sequentially run at 300 K and 1 bar using NVT and NPT ensembles to equilibrate the system. Finally, 100 ns MD simulations were performed on each molecule, producing trajectories and data files that were analyzed to elucidate protein behavior.

## Results and discussion

Twenty-four chemical descriptors and the biological activity of 32 1,2,4-triazine-3(2H)-one derivative as microtubule assembly inhibitors were used in the QSAR analysis (Table S1). The most effective correlation models were determined using the multi-linear regression technique using a set of non-collinear descriptors. Models that do not meet the OCDE standards and Golbraikh and Tropsha's requirements were disqualified^39^. The best linear model was represented by the following equation, which included the five chemical descriptors χ, TE, NHD, LogS, and I.

### MLR model for pIC50 breast cancer

18 $$\text{pIC}50 =-10.12+1.64\times\upchi -5.69{ 10}^{-05}\times \text{TE}+1.48 \times \text{NHD}-1.37\times \text{LogS}-0.36\times \text{I}$$

Since TE, LogS, and I all have negative signs in the model equation, lowering their values may raise the value of pIC50. The pIC50 value may be improved by raising X and NHD since they both have positive signs. Based on the MLR equation, it is evident that the values of the studied activity (pIC50) are linearly correlated with the five selected descriptors. As shown in Table 1, the greatest value of the coefficient of determination (R^2^ > 0.6), Furthermore, the created model is predictive, as shown by the high-value R^2^_test_. The variables were selected based on statistical significance, and we excluded variables that had high multicollinearity, as shown by the lower value of MSE and the strong statistical significance of the confidence level F (Fisher's parameter). Additionally, the robustness of the developed MLR model is shown by Q^2^'s value (more than 0.5).

**Table 1:** MLR model’s parameters.

Statistical parameter	R	R^2^	R^2^_adj_	MSE	R^2^_test_	Q^2^_cv_	F	P
Value	0.849	0.722	0.656	0.056	0.710	0.542	10.91	< 0.0001

**Figure 1:**
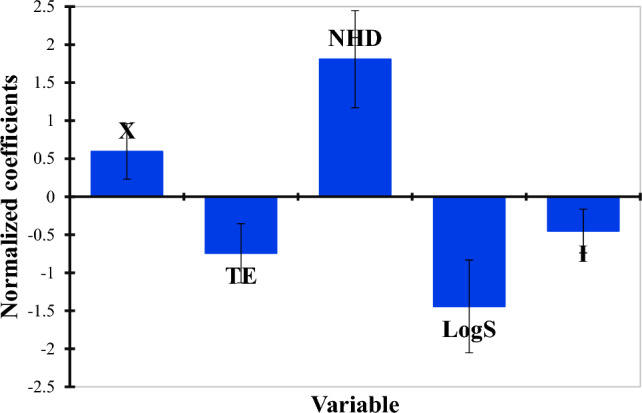
Contribution of the descriptors in model.

**Figure 2:**
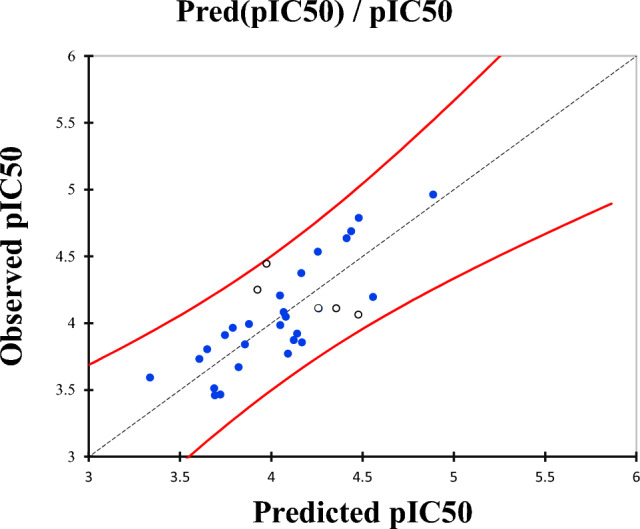
The correlation between the observed and predicted activities.

The MLR results include normalized descriptor coefficients and the correlation between observed and predicted actions (Fig. 1)^40^. The experimentally determined and theoretical pIC50 values displayed in Fig. 2 exhibit a strong correlation. The model consisted of five descriptors (Table 2) based on the normalization diagram of coefficients (Fig. 1), with NHD, LogS, TE, and X being the most significant.

**Table 2:** Chemical descriptors, observed and predicted activities, residuals using MLR model.

	No	Observed pIC50	Predicted pIC50	Residuals	*X*	TE	NHD	LogS	I
Training set	3a	3.873	4.123	− 0.250	3.69	− 22,284.29	1	− 4.24	1
	3c	4.207	4.048	0.159	3.32	− 28,517.24	1	− 4.36	1
	4a	3.965	3.789	0.177	3.55	− 30,624.97	0	− 4.63	0
	4b	3.513	3.687	− 0.174	3.33	− 33,741.52	0	− 4.69	0
	4c	3.671	3.820	− 0.150	3.26	− 36,857.93	0	− 4.74	0
	4d	3.772	4.091	− 0.319	3.26	− 39,974.39	0	− 4.81	0
	4e	3.985	4.048	− 0.063	3.28	− 43,090.46	0	− 4.63	0
	4f	3.994	3.877	0.117	3.65	− 31,695.02	0	− 4.80	1
	4g	3.805	3.648	0.157	3.35	− 34,811.42	0	− 4.86	1
	4h	3.922	4.141	− 0.219	3.28	− 37,927.91	0	− 4.91	0
	4i	4.688	4.437	0.251	3.30	− 41,044.32	0	− 4.98	0
	4j	4.636	4.412	0.224	3.33	− 44,160.43	0	− 4.79	0
	5b	3.466	3.721	− 0.255	3.34	− 31,601.86	1	− 3.97	1
	5d	4.196	4.557	− 0.361	3.32	− 37,834.79	1	− 4.09	0
	5e	3.856	4.167	− 0.311	3.34	− 40,950.81	1	− 3.91	1
	5g	4.375	4.164	0.210	3.43	− 32,671.91	1	− 4.14	1
	5h	4.110	4.259	− 0.149	3.34	− 35,788.34	1	− 4.19	1
	5i	4.963	4.886	0.076	3.34	− 38,904.72	1	− 4.26	0
	6a	4.048	4.079	− 0.031	3.70	− 27,944.84	1	− 3.70	0
	6b	3.460	3.690	− 0.230	3.51	− 31,061.38	1	− 3.77	1
	6c	3.593	3.336	0.258	3.15	− 34,177.67	1	− 3.81	1
	6e	3.733	3.606	0.127	3.19	− 40,410.15	1	− 3.70	1
	6f	4.535	4.255	0.280	3.62	− 29,014.79	1	− 3.87	0
	6g	3.911	3.746	0.165	3.37	− 32,131.27	1	− 3.94	1
	6h	3.841	3.856	− 0.015	3.29	− 35,247.71	1	− 3.98	1
	6i	4.788	4.478	0.309	3.28	− 38,364.13	1	− 4.05	0
	6j	4.083	4.067	0.016	3.30	− 41,480.16	1	− 3.87	1
Test set	3b	4.445	3.973	0.471	3.42	− 25,400.81	1	− 4.31	1
	3e	4.113	4.257	− 0.144	3.32	− 34,749.71	1	− 4.26	1
	5a	4.112	4.356	− 0.244	3.67	− 28,485.40	1	− 3.91	0
	5c	4.251	3.924	0.327	3.32	− 34,718.32	1	− 4.02	1
	5j	4.064	4.476	− 0.412	3.35	− 42,020.78	1	− 4.08	1

There is no multicollinearity among the specified descriptors, and the MLR model has strong stability, as shown by the variance inflation factor (VIF) of less than 10 for all of the created model's selected descriptors^41^. The VIF values of the derived model are shown in Table 3. Therefore, it is evident that all five of the model's descriptors have a strong correlation with pIC50. Table 2 displays the observed and predicted activity values, while their correlational relationship is illustrated in Fig. 1.

**Table 3:** The selected descriptors VIF.

Descriptors	X	TE	NHD	LogS	I
VIF	2.330	2.656	7.142	6.500	1.444

The VIF values were within an acceptable range, suggesting that multicollinearity among the selected descriptors is not a significant concern in this specific model^42^.

### Validation of the QSAR model focusing on the reliability of two-dimensional chemical descriptors

#### Y-Randomization test

A randomization test is used, and 100 models for MLR have been generated, to prevent random correlation and check the MLR model that has been built. The low values of, ${Q}_{cv \left(LOO\right)(Rand)}^{2}$ and, ${R}_{Rand}^{2}$ found for the MLR model are shown in Table S3. The findings show that the model developed is not the product of random correlation^43^.

#### Golbraikh and Tropsha criteria

The MLR model's outcomes and Golbraikh and Tropsha's parameters were compared. The findings in Table 4 demonstrate that the MLR model adheres to the standards set forth by Golbraikh and Tropsha. The model proposed is able to predict with high performance for new compounds.

**Table 4:** Comparison of model parameters (MLR) with Golbraikh and Tropsha criteria.

	Parameter	Expression	Model score	Threshold	Comment
Fitting criteria	R^2^	${R}^{2}=1-\frac{\sum {({Y}_{Obs}-{Y}_{pred})}^{2}}{\sum {({Y}_{pred}-{\overline{Y} }_{Obs})}^{2}}$	0.722	> 0.6	Passed
	R^2^_adj_	${R}_{adj}^{2}=\frac{(N-1){R}^{2}-P}{N-P-1}$	0.656	> 0.6	Passed
	MSE	$MSE=\frac{\sum ({Y}_{Obs}-{Y}_{pred})}{N}$	0.056	A low value	Passed
	F_test_	${F}_{test}=\frac{\sum {({Y}_{pred}-\overline{{Y }_{pred}})}^{2}}{\sum {({Y}_{Obs}-{Y}_{pred})}^{2}}*\frac{N-p-1}{p}$	10.91	A high value	Passed
Internal validation	Q^2^_CV_	${Q}_{CV}^{2}=1-\frac{\sum {({Y}_{Obs}-{Y^{\prime}}_{pred})}^{2}}{\sum {({Y^{\prime}}_{pred}-{\overline{Y} }_{Obs})}^{2}}$	0.542	> 0.5	Passed
	R_Rand_	Average of the 100 ${R}_{Rand}(i)$	0.438	< R	Passed
	R^2^_Rand_	Average of the 100 ${R}_{Rand}^{2}(i)$	0.208	< R^2^	Passed
	${\text{Q}}_{\text{CV LOO Rand}}^{2}$	Average of the 100 ${Q}_{CV LOO (Rand)}^{2}(i)$	− 0.331	< Q^2^_CV_	Passed
	cR^2^_p_	${cR}_{p}^{2}=R*\sqrt{({R}^{2}-{(Average {R}_{Rand})}^{2}}$	0.619	> 0.5	Passed
External validation	R^2^_test_	${R}_{test}^{2}=1-\frac{\sum {({Y}_{pred(test)}-{Y}_{Obs(test)})}^{2}}{\sum {({Y}_{Obs(test)}-{\overline{Y} }_{pred(train)})}^{2}}$	0.710	> 0.6	Passed
	$\overline{{{R }^{2}}_{m(test)}}$	$\frac{\left|{R}_{m}^{2}-{R^{\prime}}_{m}^{2}\right|}{2}$	0.530	> 0.5	Passed
	${\Delta R}_{test}^{2}$	$\left|{R}_{m}^{2}-{R^{\prime}}_{m}^{2}\right|$	0.0005	< 0.2	Passed
	${\Delta R}_{0(test)}^{2}$	$\left|{R}_{0}^{2}-{R^{\prime}}_{0}^{2}\right|$	0.001	< 0.3	Passed
	k	$k=\frac{\sum {Y}_{obs}{Y}_{pred}}{\sum {Y}_{pred}^{2}}$	0.996	$0.85 \le \text{k}\le 1.15$	Passed
	k’	$k^{\prime}=\frac{\sum {Y}_{obs}{Y}_{pred}}{\sum {Y}_{obs}^{2}}$	0.997	$0.85 \le \text{k}^{\prime}\le 1.15$	Passed

#### Applicability domain

**Figure 3:**
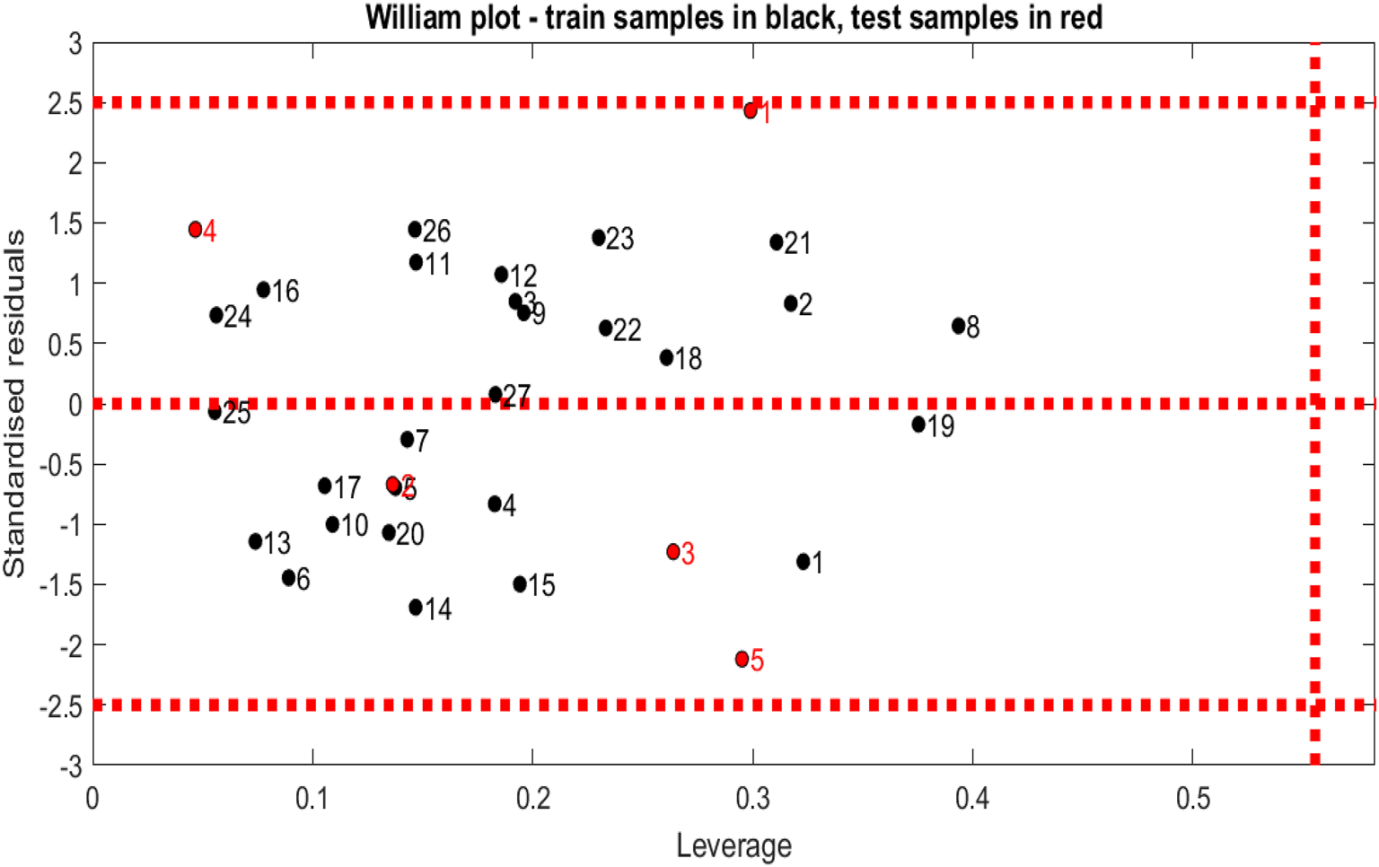
Williams plot of standardized residual versus leverage for the MLR model (with: h* = 0.56 and residual limits =  ± 2.5); training samples in the black color and test samples in the red color.

By plotting the leverage effect values (hi) vs the residual values, the application domain of the verified model was identified. The distribution of normalized residual values, the leverage level values, and the threshold leverage effect (h* = 0.56), with ${h}^{*}=3\times \frac{k+1}{n}$; k = 5; N = 32, as well as the distribution of normalized residual values and the leverage level values have calculated as shown in following William’s plot (Fig. 3). Based on the levers (h* > 0.56), none of the compounds was found outside the AD of the developed model.

Using MatlabV2021a software, we define the Williams plot type's application domain in this study.

### Design of new compounds

The suggested model demonstrates how adjustments to the QSAR model's appeared descriptors might improve activity. The finished model's descriptors, on the other hand, may be used to interpret the data. Finding the connection between the descriptor values and the researched compounds' structural specifics is simple.

#### Absolute electronegativity (χ)

This descriptor represents the tendency of a molecule to attract electrons. In our QSAR model, a higher value of χ corresponds to increased electron-withdrawing capacity, which enhances the interaction of the molecule with the target protein, thereby increasing the pIC50 values. This suggests that molecules with higher electronegativity are more effective in disrupting the Tubulin dynamics critical for cancer cell viability.

#### Total energy (TE)

The total energy of a molecule reflects its stability; in our study, a lower TE is associated with higher biological activity. Molecules with lower total energy are more stable and can more readily interact with the target site on the Tubulin protein, which could explain their enhanced inhibitory effects on breast cancer cell lines.

#### Number of hydrogen bond donors (NHD)

Hydrogen bond donors play a crucial role in drug-target interactions by forming hydrogen bonds with the target protein, stabilizing the drug-protein complex. Our findings indicate that an increase in NHD enhances pIC50, suggesting that additional hydrogen bonds contribute positively to the binding affinity and thus the effectiveness of the inhibition.

#### Water solubility (LogS)

Solubility is critical for the bioavailability of a drug. In our model, compounds with better solubility (higher LogS values) generally exhibited lower pIC50, indicating that while solubility is crucial for overall drug performance, excessively soluble compounds may be too hydrophilic to efficiently permeate cell membranes and reach intracellular targets.

#### Shape coefficient (I)

This descriptor quantifies the three-dimensional shape and bulk of the molecule, influencing how well the molecule fits into the target binding site. Our results suggest that a smaller shape coefficient, indicating a more compact molecular structure, is beneficial for interacting with the specific contours of the Tubulin binding site, thereby enhancing inhibitory activity.

As a result, we decided to search for novel compounds with specific alterations in the structural characteristics of the compounds under study. The impact of descriptors on the biological activity and structural characteristics of the most active compounds led to the suggestion of several compounds. ChemOffice software was used to sketch and optimize the structures of the suggested compounds, and ChemOffice and Gaussian software was used to determine the effective descriptors.

The structures of the proposed compounds and their corresponding predicted pIC50 values are summarized in Tables S4 and 5. The data indicate that increases in the values of X and NHD enhance the anti-cancer activity of the dataset compounds, whereas increases in the values of TE, LogS, and I diminish this activity. The addition of elements with greater electronegativity than carbon (e.g., F, Cl, O, and N) and an increase in molecular size lead to an increase in the value of X and a decrease in the value of the descriptor TE. Based on this information, modifications were made to the 1,2,4-triazin-3(2H)-one ring in the template compound 5i. This phase involved the design of 28 compounds and the calculation of their leverage values to identify outliers. Equation (18) was utilized to determine the pIC50 values. Tables S4 and 5 present the structures, values of chemical descriptors, and pIC50 values of these compounds.

**Table 5:** The values of chemical descriptors, the predicted activities using the MLR model.

Compound	*X*	TE	NHD	LogS	I	pIC50
Pred1	3.59	− 38,488.55	1	− 4.47	0	5.56
Pred2	3.75	− 48,294.76	1	− 4.88	0	6.95
Pred3	3.75	− 38,072.44	1	− 4.68	0	6.09
Pred4	3.56	− 38,072.37	1	− 4.69	1	5.43
Pred5	3.91	− 37,656.22	1	− 4.90	0	6.62
Pred6	3.46	− 105,753.16	1	− 5.01	0	9.91
Pred7	3.67	− 36,349.39	3	− 4.23	0	8.21
Pred8	3.72	− 41,749.65	3	− 4.70	0	9.24
Pred9	3.86	− 42,061.89	1	− 4.77	0	6.62
Pred10	3.51	− 35,372.05	1	− 4.41	1	4.81
Pred11	4.02	− 35,754.97	1	− 4.06	0	5.54
Pred12	3.92	− 41,571.56	1	− 4.33	0	6.08
Pred13	3.88	− 44,687.87	1	− 4.16	0	5.97
Pred14	3.45	− 41,604.75	1	− 4.25	1	4.86
Pred15	3.51	− 35,372.05	1	− 4.39	1	4.79
Pred16	2.90	− 46,999.25	2	− 4.76	1	6.43
Pred17	3.30	− 51,410.93	1	− 4.94	0	6.48
Pred18	2.98	− 40,411.16	2	− 4.06	0	5.61
Pred19	3.14	− 40,411.30	2	− 4.11	0	5.93
Pred20	3.17	− 40,411.17	2	− 4.06	0	5.91
Pred21	3.14	− 35,684.73	3	− 3.86	1	6.44
Pred22	3.03	− 34,074.65	4	− 3.66	0	7.74
Pred23	3.21	− 34,074.57	4	− 3.60	0	7.96
Pred24	3.07	− 35,580.95	5	− 3.19	0	8.73
Pred25	3.04	− 37,087.31	6	− 2.77	0	9.68
Pred26	3.42	− 39,994.98	2	− 4.29	0	6.61
Pred27	3.81	− 43,131.76	1	− 5.13	0	7.10
Pred28	3.89	− 42,641.58	1	− 4.53	0	6.38

### Drug‑likeness assessment and ADMET predictions

The physicochemical characteristics of the substances are often linked to certain filter variations when assessing how drug-like they are. So, via the ADMETlab 2.0 Web server, pertinent physicochemical parameters (Table S5) are produced. According to the radar charts, the physicochemical characteristics of the fourteen compounds fall between the upper (brown) and lower (red) bounds (Table S5). The fourteen compounds (Table S6) that passed the Lipinski rule of five were further evaluated using the SwissADME Web server utilizing various drug-likeness filter rules, including the Ghose filter rule, Veber's rule, and Egan's rule. The results are reported in Table S6.

Using Lipinski's rule of five as a filter, the SwissADME web server was used to theoretically forecast the drug-like characteristics of the examined compounds (Table S6), including the best-hit compounds. Any small molecule that fails to meet more than one of the aforementioned requirements may have problems with bioavailability. The novel compounds' pharmacokinetic analysis is shown in Table S6, and All of the compounds pass the Lipinski rule of five tests, including the criterion for molecular weight, which stipulates a maximum of 500 g/mol. Furthermore, all of the best hit compounds had one hydrogen bond acceptors, which was within the accepted range. For the number of hydrogen bond donors, between 3 and 8 is also within the accepted range. Additionally, the estimated LogP value never went beyond or below the acceptable bounds. The best-hit compounds were determined to be drug-like based on these filtering requirements by not exceeding the established threshold values (or by not having more than one violation of the filtering conditions used). They were all further determined to be orally bioavailable based on the value of their bioavailability score (all having 0.56)^26^. Given that these substances complied with all of the filtering requirements, it was generally anticipated that there would be no problems with their bioavailability.

The examined compounds, including the top hit compounds, were theoretically evaluated for their ADMET/pharmacokinetic characteristics using the online web server pkCSM (Table S7). Human intestinal absorption of the best-identified hit compounds was all found to vary between 63.568 and 96.568%. These small molecules can be absorbed inside the human gut if their values of intestinal absorption in humans are larger than the minimum suggested rate of 30% given for the assessment of this attribute. The accepted threshold value for the central nervous system (CNS) permeability is > − 2 to < − 3, while that for the blood–brain barrier (BBB) permeability is > − 0.3 to < − 1. The BBB permeability for these small molecules was found to be − 1 except for compounds Pred9, Pred10, Pred11, Pred15, Pred27, and Pred28, which was > − 1. This indicates that all of the compounds, except for compounds Pred9, Pred10, Pred11, Pred15, Pred27, and Pred28, only partially infiltrate or permeate the BBB. Except for compounds Pred2, Pred9, Pred10, Pred11, and Pred27, all of the compounds had a CNS permeability value of < − 3, indicating that they only partially infiltrate or permeate the CNS.

The cytochrome (CYP) is recognized as being crucial for the body's enzymatic breakdown and small molecule metabolism. As a result, it is important to consider how these small molecules are metabolized and processed by the human body. The body's mechanisms for breaking down and metabolizing small compounds include CYP1A2, 2C9, 2C19, 2D6, and 3A4, with CYP3A4 playing the most important role (a good small molecule is expected to be both a substrate and inhibitor of CYP3A4)^26^. The most popular hits were all CYP3A4 substrates and inhibitors. This led to more confirmation that the body can break down these small compounds. Excretion/total clearance, which specifies the connection/relationship between the rate of these small molecules' elimination and concentration inside the body, is another crucial consideration. The best-hit compounds showed a higher excretion value and were tested within the acceptable range for a medication. The comprehensive toxicity and pharmacokinetic evaluation detailed in Table S7 reveals a mixed safety profile for the best-identified hit drugs. While none of the compounds displayed mutagenicity, suggesting a low risk of genetic toxicity which bolsters their overall safety profiles, several compounds, specifically Pred1, Pred2, Pred3, Pred4, Pred10, Pred12, Pred14, and Pred15, exhibited potential hepatotoxic effects. These findings necessitate further in-depth investigations, including in vitro and in vivo studies, to thoroughly understand the implications for liver health and to mitigate any adverse effects. Moreover, only Pred11 was flagged for potential carcinogenic risks, indicating a need for extended research and evaluation through long-term animal studies to ascertain the full extent of this risk. Despite these concerns, the broad ADMET properties of these compounds were typical, aligning with expected pharmacokinetic profiles and confirming that none were overtly harmful.

### Molecular docking study

An Intel (R) core (TM) i5 10th Gen processor-equipped microcomputer was used to carry out the computations for molecular docking. The operating system for all apps was Windows 10, 64-bit version 2020. The software AutoDockTools version 1.5.7 was used to carry out the molecular docking^33^, this employs the Genetic algorithm-based trajectory modeling technique. Additionally, we used Biovia Discovery Studio version 2021 software^44^ to display the many interactions that have been created between the ligands and the target active site.

**Figure 4:**
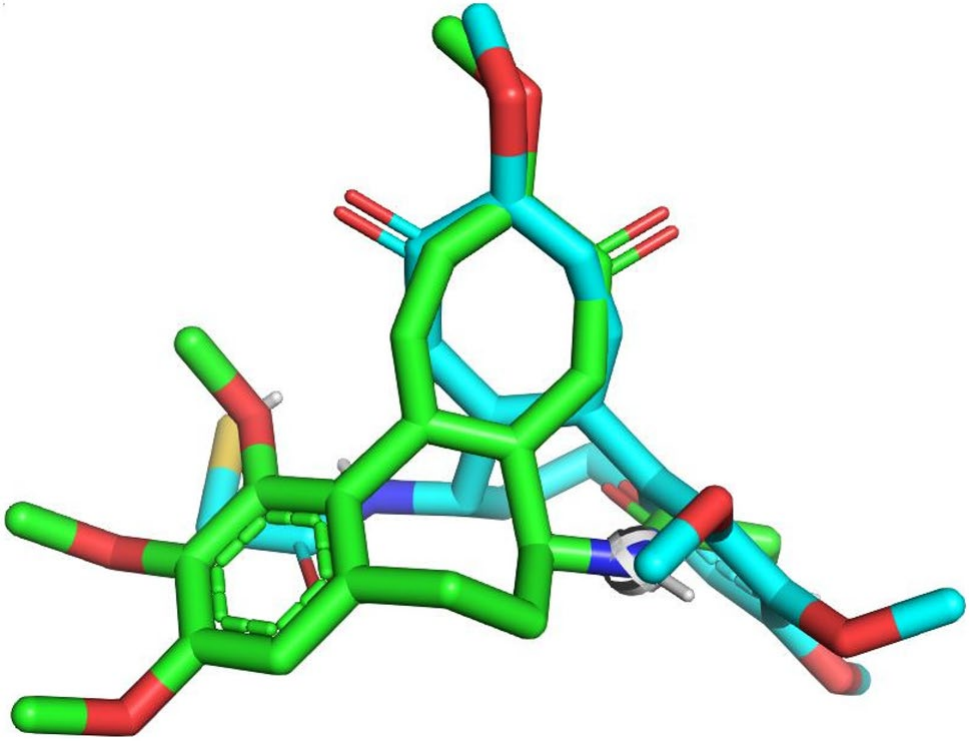
Re-docking pose with an RMSD value of 1.084 Å (Green = native ligand, blue = docked ligand).

Molecular docking has become an increasingly popular approach for the development of new drugs, in part because of the favorable time and pecuniary costs of in silico drug screening compared with traditional laboratory experiments. In this study, the fourteen designed compounds were docked, using AutoDock 1.5.7 software, with the crystal structure 1SA0 of the target protein’s binding site^31^. To perform docking by re-docking the co-crystallized ligand at the Tubulin enzyme Fig. 4 represented the superimposed view of docked conformation^45–49^ and the co-crystallized ligand and the RMSD value is 1.084 Å. The RMSD (Root Mean Square Distance) of the docked co-crystallized ligand was within the reliable range of 2 Å. The binding affinity of the designed compounds with the receptors 1SA0 was reported in Table 6.

**Table 6:** Results of the binding affinity of the most stable conformation.

Compound	Binding affinity (kcal/mol)
Co-crystallized ligand	− 7.9
Pred1	− 8.9
Pred2	− 8.5
Pred3	− 9.2
Pred4	− 8.9
Pred5	− 9.1
Pred9	− 8.6
Pred10	− 8.8
Pred11	− 8.7
Pred12	− 9.0
Pred13	− 9.2
Pred14	− 8.7
Pred15	− 8.7
Pred27	− 8.9
Pred28	− 9.6

#### Microtubule protein A and B chains: structural and functional insights

Microtubules, essential components of the cytoskeleton, are composed of α-Tubulin (A chain) and β-Tubulin (B chain) that form heterodimers. α-Tubulin forms the stable minus end of the microtubule and harbors a non-exchangeable GTP molecule, providing structural stability. In contrast, β-Tubulin constitutes the dynamic plus end, featuring an exchangeable GTP site crucial for microtubule dynamics through GTP hydrolysis post-polymerization. This differential GTP activity between the chains underpins the dynamic instability vital for cellular processes such as mitosis and intracellular transport. Given β-Tubulin's pivotal role in these processes and its drug target accessibility, our study focuses on inhibitors that target β-Tubulin to disrupt microtubule dynamics, offering potential therapeutic strategies against rapidly dividing cancer cells.

All the designed compounds exhibited docking score values between − 8.5 and − 9.6 kcal/mol, having lower binding energies than the co-crystallized ligand -7.9 kcal/mol. The compounds Pred3, Pred5, Pred12, Pred13 and Pred28 showed the best binding score with the protein Tubulin. The best conformation from Autodock resulted with good binding affinity of compound Pred28 with 1SA0 protein as   − 9.6 kcal/mol, based on Table S8 and Fig. S1, has six conventional hydrogen bonds interactions with Lys (B: 254), Gly (A: 146), Gly (A: 142), Thr (A: 145), Gln (A: 11), Asn (A: 101), and H-donor, two carbon hydrogen bonds with Lys (B: 254) and Gln (A: 11), two halogen type fluorine bonds with Glu (A: 183) and Glu (A: 142), hydrophobic interactions with Lys (B: 254), Ala (A: 12), Ile (A: 171), Pro (A: 173), and Leu (B: 248) residues.

The compound Pred13 formed six conventional hydrogen bonds interactions with Gly (A: 144), Asn (A: 101), Asp (A: 69), Gly (A: 142), Lys (B: 254), and Thr (A: 145), two carbon hydrogen bonds with Lys (B: 254) and Gln (A: 11), one halogen bond with Glu (A: 183), it exhibited also hydrophobic interactions with Tyr (A: 224), Lys (B: 254), Leu (B: 248), Ala (A: 12), Ile (A: 171), and Pro (A: 173) residues.

The compound Pred12 formed five conventional hydrogen bonds interactions with Gly (A: 146), Asn (A: 101), Thr (A: 145), Gln (A: 11), and Ala (A: 12), one carbon hydrogen bond with Gln (A: 11), hydrophobic interactions with Ala (A: 12), Tyr (A: 224), Leu (B: 248), Ala (A: 12), and Ile (A: 171) residues.

The compound Pred5 formed five conventional hydrogen bonds interactions with Gly (A: 146), Asn (A: 101), Tyr (A: 224), Ser (A: 140), and Lys (B: 254), one carbon hydrogen bond Pro (A: 173), one halogen bond with Asn (A: 206), it exhibited also hydrophobic interactions with Gly(A: 143), Gly (A: 10), and Ala (A: 12) residues.

The compound Pred3 formed six conventional hydrogen bonds interactions with Gly (A: 146), Asn (A: 101), Tyr (A: 224), Glu (A: 183), Lys (B: 254), and Ser (A: 140), three carbon hydrogen bonds with Pro (A: 173), Glu (A: 71), and Asp (A: 98), two halogen bonds with Asn (A: 206) and Tyr (A: 172), hydrophobic interactions with Gly (A: 143), Gly (A: 10), and Ala (A: 12) residues.

The compound co-crystallized ligand formed two carbon hydrogen bonds interactions with Ser (A: 178) and Val (B: 315), one Pi-Sulfur bond with Met (B: 259), hydrophobic interactions with Ala (B: 316), Ala (A: 180), Lys (B: 352), Leu (B: 248), Ala (A: 180), and Lys (B: 352) residues.

A significant aspect of our study lies in correlating the molecular descriptors used in the QSAR model with the binding modes observed in the docking studies. For example, the descriptor 'χ' (absolute electronegativity) correlates with the affinity of the ligand for the negatively charged regions of the protein active site. On the other hand, 'LogS' (solubility log) is reflective of the hydrophobic interactions that are crucial for the stability of the ligand–protein complex. Thus, each descriptor serves not only as a statistical variable but also as a biologically relevant parameter that can be linked to specific aspects of ligand–protein interactions.

We fully acknowledge that while computational methods like QSAR modeling and molecular docking are valuable tools for predicting the biological activity of compounds, they are not a substitute for experimental validation. In an ideal scenario, the next step would involve the chemical synthesis and experimental testing of the predicted 1,2,4-triazine-3(2H)-one derivatives. Due to limitations in scope and resources, this was not possible within the confines of the present study. However, we strongly advocate for such experimental validation in future work to confirm the predicted activities of these promising compounds.

### Molecular dynamic simulation

In this study, Molecular Dynamics Simulations (MDS) were conducted for 100 ns to investigate the binding interactions, structural dynamics, and flexibility of Tubulin when bound to the top three hits (Pred3, Pred13, and Pred28). Key metrics, including Root Mean Square Deviation (RMSD), Root Mean Square Fluctuation (RMSF), and Radius of Gyration (Rg), were calculated from the trajectory data collected over the 100-ns period^50^.

#### RMSD calculation

**Figure 5:**
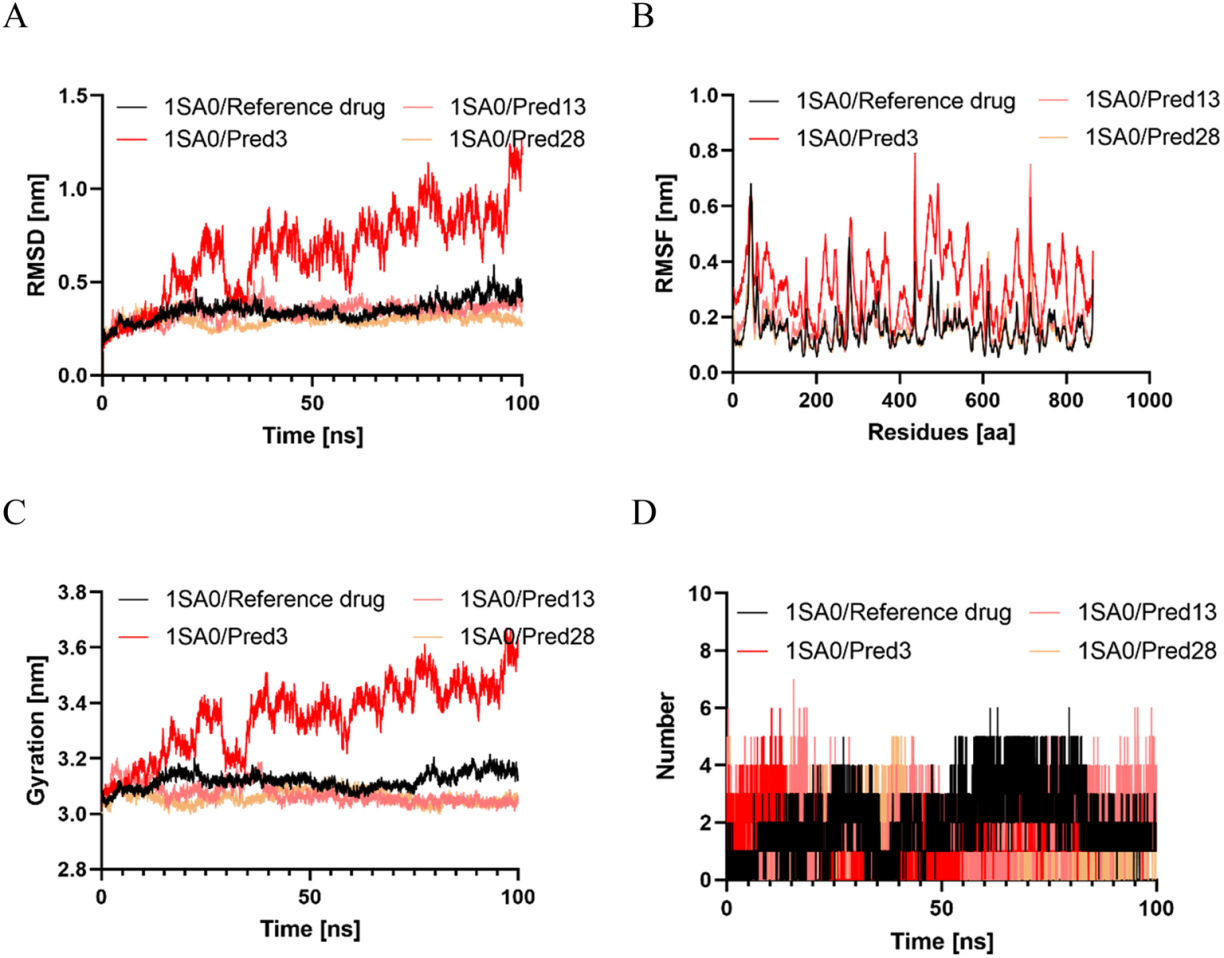
The results of the molecular dynamics study: (**A**) Time evolution of the backbone of three best hits inhibitors and the standard (Colchicine); (**B**); RMSF spectra of three promising inhibitors and the standard (Colchicine); (**C**) The comparative Radius of gyration values for the target protein with three best hits inhibitors and the standard (Colchicine); (**D**) The comparative hydrogen bonds for the target protein with the reference drug (Colchicine) and three best hits.

The root mean square deviation (RMSD) is the most important indicator of a biomolecular system's structural stability. The computer-aided drug design community believes that a lower RMSD value signifies a more stable system. In contrast, complexes with larger RMSD values are less likely to be stable^51^. The RMSD graph in Fig. 5A shows that, except for the complex formed by Pred3-Tubulin, all the remaining protein–ligand entities exhibit lower fluctuations in their spectra. This implies that their conformational dynamics have been minimally perturbed during the simulation. The average RMSD values for Pred3, Pred13, Pred28, and Colchicine (with their receptors) are calculated to be 0.65 nm, 0.34 nm, 0.29 nm, and 0.34 nm, respectively. This indicates that Pred28, with its lower RMSD value, is most similar to the standard Colchicine drug and may represent the most stable drug candidate among the promising inhibitors. This finding aligns with the docking simulation results, where Pred28 exhibited the best binding affinity (Table 6). However, the RMSD measurements are insufficient to ensure the stability of the system because they do not account for fluctuations in certain sections of the structure. Therefore, an RMSF diagram was also constructed to evaluate the atomic variations of each residue along the trajectory.

#### RMSF calculation

For a comprehensive description of residue stability in MD simulation, RMSF measurement is the gold standard^52^. In the simulation, this approach assesses protein flexibility and can identify residues with high or low flexibility. Root-mean-square-fluctuations (RMSF) were calculated for each complex to reveal which residues changed the most during the MD simulation^47,53^. Higher RMSF values for biomolecular system corresponds to a lower residual stability and vice versa. As demonstrated in Fig. 5B, except the Pred3, all the remaining proposed compound exhibit a very similar RMSF pattern to the standard drug. The average RMSF values are 0.31 nm for Pred3, 0.16 nm for Pred13, 0.15 nm for Pred28, and 0.15 nm for Colchicine. Notably, Pred28, which has the lowest average RMSF value, indicates it may be a better inhibitor compared to the other compounds.

#### Radius of gyration analysis

We investigated how the binding of various ligands influenced the overall compactness of the protein's structure^48^. To achieve this, we calculated the radius of gyration (Rg) as a function of time. Ligands with higher Rg values tend to be more flexible and, consequently, less stable. Conversely, lower Rg values indicate a denser and more compact conformation^38^. Among the complexes examined (Fig. 5C), the Pred3 exhibited the largest radius of gyration, while compound Pred28 had the smallest, indicating it is the most stable. The average Rg values for protein-Reference drug complexe and protein-designed molecule complexes (including Pred03, Pred13, and Pred28) were 3.11 nm, 3.35, 3.07 nm, and 3.06 nm, respectively. This suggests that complex Pred28 is the most the most compact biomolecular system.

#### H-BOND analysis

In a water environment, hydrogen bonds and their relative strength play a crucial role in protein–ligand interactions, especially when the mechanism involves hydrolysis, where water is essential for breaking down a chemical^54^. These hydrogen bonds form when an electronegative atom from a hydrogen-bond acceptor comes into contact with a hydrogen atom directly bonded to an electronegative atom from a hydrogen-bond donor^55^.

In this study, we examined the intermolecular hydrogen bonds formed between Tubulin and the selected compounds (Pred3, Pred13, Pred28, and Colchicine). The results, presented in Fig. 5D, show that Pred28 and Colchicine have the most significant H-bond spectra among all the simulated compounds, with average H-bonds of 1.75 and 1.94, respectively. In contrast, Pred3 and Pred13 formed fewer intermolecular H-bonds with their receptor, averaging 1.37 and 1.02 H-bonds, respectively. This finding aligns with earlier RMSD and RMSF analyses, further supporting that Pred28 is the best compound, as the H-bonding spectrum results corroborate this conclusion.

## Conclusion

In this study, we have demonstrated the potential of 1,2,4-triazine-3(2H)-one derivatives as effective Tubulin inhibitors through a comprehensive application of QSAR modeling, molecular docking, and molecular dynamics simulations. These findings not only enhance our understanding of the chemical interactions necessary for Tubulin inhibition but also highlight the robust potential of these compounds to serve as templates for developing new anticancer drugs. The integration of multiple computational techniques has allowed for a nuanced assessment of the compounds' interactions with Tubulin, revealing that among the tested derivatives, Pred28 emerged as a particularly promising candidate due to its stability and strong binding affinity. Our study extends beyond the conventional discovery phase to provide a detailed characterization of the dynamic interactions and stability profiles of these inhibitors, setting the stage for future in vitro and in vivo evaluations. Furthermore, our research contributes to the ongoing efforts to optimize anticancer therapies by providing a clearer pathway for the rational design of drug candidates. By identifying and characterizing novel inhibitors that can potentially overcome resistance mechanisms or reduce adverse effects associated with current treatments, this work paves the way for more targeted and effective cancer treatment strategies. Looking forward, the promising results obtained from Pred28 warrant further investigation to confirm its therapeutic value and explore its efficacy in clinical settings. Additionally, the methodologies applied in this study can be adapted to other therapeutic targets and diseases, underscoring the versatility and impact of computational approaches in modern drug discovery. In conclusion, the findings from this study not only advance our knowledge of the molecular underpinnings of Tubulin inhibition but also provide a solid foundation for the future development of more effective and safer anticancer therapies. As we continue to explore these novel compounds, it is our hope that they will contribute to a more effective arsenal against cancer, ultimately improving patient outcomes.

### Supplementary Information

Supplementary Information 1.

Supplementary Information 2.

## Data Availability

All available data can be found within the article and in the supplementary file. Further inquiries can be directed to the corresponding author.
