# Supplementary material for: QSAR, ADMET, molecular docking, and dynamics studies of 1,2,4-triazine-3(2H)-one derivatives as tubulin inhibitors for breast cancer therapy
Source: Sci Rep. 2024 Jul 16;14:16418. doi: 10.1038/s41598-024-66877-2 (PMC11252338; doi:10.1038/s41598-024-66877-2)
Supplement: Supplementary file 1 — Supplementary Information 1. [file 41598_2024_66877_MOESM1_ESM.docx]

QSAR, ADMET, Molecular Docking, and Dynamics Studies of 1,2,4-Triazine-3(2H)-one Derivatives as Tubulin Inhibitors for Breast Cancer Therapy

Mohamed Moussaoui^1*^, Soukayna Baammi^2^, Hatim Soufi^1^, Mouna Baassi^1^, Achraf EL Allali^2^, M.E. Belghiti^1,3^, Rachid Daoud^4*^, Said Belaaouad^1^

^1^Laboratory of Physical Chemistry of Materials, Faculty of Sciences Ben M’Sick, Hassan II University of Casablanca, Morocco.

^2^Bioinformatics Laboratory, College of Computing, Mohammed VI Polytechnic University, Ben Guerir, Morocco

^3^Laboratory of Nernest Technology, 163 Willington Street, Sherbrook, J1H5C7, Quebec, Canada.

^4^Chemical and Biochemical Sciences-Green Processing Engineering, Mohammed VI Polytechnic University, Ben Guerir, Morocco

* Corresponding author: Mohamed Moussaoui ([moussaouimohamed143@gmail.com](mailto:moussaouimohamed143@gmail.com))

* Corresponding author: Rachid Daoud ([rachid.daoud@um6p.ma](mailto:rachid.daoud@um6p.ma))

| **Tables and Figure of contents** | **Pages** |
| --- | --- |
| **Table S1.** pIC50 values of the reported 1,2,4-triazin-3(2H)-ones derivatives against targeting colchicine | **S2** |
| **Table S2.** Correlation matrix between different obtained descriptors | **S4** |
| **Table S3.** Results of Y randomization tests for model | **S5** |
| **Table S4.** Calculated anticancer activity and leverages (h) for the new designed 1,2,4-triazin-3(2H)-ones derivatives | **S6** |
| **Table S5.** Physico-Chemical radar chart of the selected lead compounds in the dataset | **S7** |
| **Table S6.** Lipinski-Veber-Egan properties of the new designed 1,2,4-triazin-3(2H)-ones derivatives | **S9** |
| **Table S7.** ADMET/pharmacokinetic features of the best-hit compounds | **S10** |
| **Figure S1.** 3D and 2D representations of the binding interactions of the 15 complex compounds | **S11** |
| **Table S8.** Interaction table between the compounds and 1SA0 protein cancer | **S19** |

**Table S1.** pIC50 values of the reported 1,2,4-triazin-3(2H)-ones derivatives against targeting colchicine

| **Training/Test** | **Compounds** | **N** | **R1** | **R2** | **R3** | **R4** | **pIC50** |
| --- | --- | --- | --- | --- | --- | --- | --- |
| **Training** |  | **3a** | **H** | **H** | **H** | **H** | **3.873** |
| **Test** |  | **3b** | **OCH_3_** | **H** | **H** | **H** | **4.445** |
| **Training** |  | **3c** | **OCH_3_** | **H** | **OCH_3_** | **H** | **4.207** |
| **Test** |  | **3e** | **OCH_3_** | **OCH_3_** | **OCH_3_** | **OCH_3_** | **4.113** |
| **Training** |  | **4a** | **H** | **H** | **H** | **H** | **3.965** |
| **Training** |  | **4b** | **OCH_3_** | **H** | **H** | **H** | **3.513** |
| **Training** |  | **4c** | **OCH_3_** | **H** | **OCH_3_** | **H** | **3.671** |
| **Training** |  | **4d** | **OCH_3_** | **OCH_3_** | **H** | **OCH_3_** | **3.772** |
| **Training** |  | **4e** | **OCH_3_** | **OCH_3_** | **OCH_3_** | **OCH_3_** | **3.985** |
| **Training** |  | **4f** | **H** | **H** | **H** | **H** | **3.994** |
| **Training** |  | **4g** | **OCH_3_** | **H** | **H** | **H** | **3.805** |
| **Training** |  | **4h** | **OCH_3_** | **H** | **OCH_3_** | **H** | **3.922** |
| **Training** |  | **4i** | **OCH_3_** | **OCH_3_** | **H** | **OCH_3_** | **4.688** |
| **Training** |  | **4j** | **OCH_3_** | **OCH_3_** | **OCH_3_** | **OCH_3_** | **4.636** |
| **Test** |  | **5a** | **H** | **H** | **H** | **H** | **4.112** |
| **Training** |  | **5b** | **OCH_3_** | **H** | **H** | **H** | **3.466** |
| **Test** |  | **5c** | **OCH_3_** | **H** | **OCH_3_** | **H** | **4.251** |
| **Training** |  | **5d** | **OCH_3_** | **OCH_3_** | **H** | **OCH_3_** | **4.196** |
| **Training** |  | **5e** | **OCH_3_** | **OCH_3_** | **OCH_3_** | **OCH_3_** | **3.856** |
| **Training** |  | **5g** | **OCH_3_** | **H** | **H** | **H** | **4.375** |
| **Training** |  | **5h** | **OCH_3_** | **H** | **OCH_3_** | **H** | **4.110** |
| **Training** |  | **5i** | **OCH_3_** | **OCH_3_** | **H** | **OCH_3_** | **4.963** |
| **Test** |  | **5j** | **OCH_3_** | **OCH_3_** | **OCH_3_** | **OCH_3_** | **4.064** |
| **Training** |  | **6a** | **H** | **H** | **H** | **H** | **4.048** |
| **Training** |  | **6b** | **OCH_3_** | **H** | **H** | **H** | **3.460** |
| **Training** |  | **6c** | **OCH_3_** | **H** | **OCH_3_** | **H** | **3.593** |
| **Training** |  | **6e** | **OCH_3_** | **OCH_3_** | **OCH_3_** | **OCH_3_** | **3.733** |
| **Training** |  | **6f** | **H** | **H** | **H** | **H** | **4.535** |
| **Training** |  | **6g** | **OCH_3_** | **H** | **H** | **H** | **3.911** |
| **Training** |  | **6h** | **OCH_3_** | **H** | **OCH_3_** | **H** | **3.841** |
| **Training** |  | **6i** | **OCH_3_** | **OCH_3_** | **H** | **OCH_3_** | **4.788** |
| **Training** |  | **6j** | **OCH_3_** | **OCH_3_** | **OCH_3_** | **OCH_3_** | **4.083** |

**Table S2.** Correlation matrix between different obtained descriptors

**Table S3.** Results of Y randomization tests for model

| **Rand** | **R_Rand_** | **R^2^_Rand_** | **Q^2^_cv (Rand)_** | **Rand** | **R_Rand_** | **R^2^_Rand_** | **Q^2^_cv (Rand)_** | **Rand** | **R_Rand_** | **R^2^_Rand_** | **Q^2^_cv (Rand)_** | **Rand** | **R_Rand_** | **R^2^_Rand_** | **Q^2^_cv (Rand)_** |
| --- | --- | --- | --- | --- | --- | --- | --- | --- | --- | --- | --- | --- | --- | --- | --- |
| **1** | 0.531 | 0.282 | -0.343 | **26** | 0.409 | 0.167 | -0.374 | **51** | 0.639 | 0.408 | 0.045 | **76** | 0.339 | 0.115 | -0.379 |
| **2** | 0.250 | 0.063 | -0.594 | **27** | 0.673 | 0.454 | 0.109 | **52** | 0.364 | 0.133 | -0.325 | **77** | 0.499 | 0.249 | -0.311 |
| **3** | 0.186 | 0.035 | -0.500 | **28** | 0.401 | 0.161 | -0.553 | **53** | 0.601 | 0.362 | -0.023 | **78** | 0.479 | 0.229 | -0.251 |
| **4** | 0.600 | 0.360 | -0.001 | **29** | 0.590 | 0.349 | -0.032 | **54** | 0.724 | 0.524 | 0.246 | **79** | 0.507 | 0.257 | -0.277 |
| **5** | 0.538 | 0.289 | -0.271 | **30** | 0.346 | 0.120 | -0.380 | **55** | 0.451 | 0.204 | -0.375 | **80** | 0.570 | 0.325 | -0.065 |
| **6** | 0.478 | 0.228 | -0.523 | **31** | 0.282 | 0.080 | -0.764 | **56** | 0.355 | 0.126 | -0.681 | **81** | 0.440 | 0.193 | -0.304 |
| **7** | 0.345 | 0.119 | -0.391 | **32** | 0.368 | 0.136 | -0.396 | **57** | 0.467 | 0.218 | -0.379 | **82** | 0.452 | 0.204 | -0.312 |
| **8** | 0.516 | 0.266 | -0.319 | **33** | 0.475 | 0.226 | -0.222 | **58** | 0.322 | 0.104 | -0.471 | **83** | 0.418 | 0.175 | -0.281 |
| **9** | 0.450 | 0.202 | -0.347 | **34** | 0.417 | 0.174 | -0.334 | **59** | 0.676 | 0.458 | 0.143 | **84** | 0.483 | 0.233 | -0.279 |
| **10** | 0.467 | 0.218 | -0.203 | **35** | 0.425 | 0.181 | -0.457 | **60** | 0.201 | 0.041 | -0.554 | **85** | 0.305 | 0.093 | -0.444 |
| **11** | 0.549 | 0.301 | -0.222 | **36** | 0.425 | 0.181 | -0.333 | **61** | 0.490 | 0.240 | -0.363 | **86** | 0.346 | 0.120 | -0.531 |
| **12** | 0.365 | 0.133 | -0.383 | **37** | 0.414 | 0.172 | -0.424 | **62** | 0.579 | 0.336 | -0.186 | **87** | 0.330 | 0.109 | -0.330 |
| **13** | 0.441 | 0.195 | -0.229 | **38** | 0.077 | 0.006 | -0.561 | **63** | 0.536 | 0.287 | -0.219 | **88** | 0.523 | 0.274 | -0.188 |
| **14** | 0.368 | 0.136 | -0.338 | **39** | 0.505 | 0.255 | -0.190 | **64** | 0.264 | 0.070 | -0.471 | **89** | 0.307 | 0.094 | -0.616 |
| **15** | 0.606 | 0.367 | -0.019 | **40** | 0.475 | 0.225 | -0.228 | **65** | 0.578 | 0.334 | -0.063 | **90** | 0.413 | 0.170 | -0.520 |
| **16** | 0.528 | 0.279 | -0.276 | **41** | 0.613 | 0.375 | -0.095 | **66** | 0.290 | 0.084 | -0.513 | **91** | 0.418 | 0.175 | -0.405 |
| **17** | 0.496 | 0.246 | -0.245 | **42** | 0.212 | 0.045 | -0.775 | **67** | 0.343 | 0.118 | -0.574 | **92** | 0.426 | 0.181 | -0.342 |
| **18** | 0.386 | 0.149 | -0.392 | **43** | 0.465 | 0.216 | -0.322 | **68** | 0.622 | 0.387 | 0.060 | **93** | 0.733 | 0.538 | 0.205 |
| **19** | 0.163 | 0.026 | -0.781 | **44** | 0.384 | 0.147 | -0.669 | **69** | 0.606 | 0.367 | 0.015 | **94** | 0.501 | 0.251 | -0.115 |
| **20** | 0.564 | 0.318 | -0.119 | **45** | 0.362 | 0.131 | -0.713 | **70** | 0.447 | 0.200 | -0.334 | **95** | 0.140 | 0.020 | -0.834 |
| **21** | 0.323 | 0.104 | -0.549 | **46** | 0.530 | 0.281 | -0.152 | **71** | 0.325 | 0.106 | -0.488 | **96** | 0.367 | 0.135 | -0.406 |
| **22** | 0.500 | 0.250 | -0.210 | **47** | 0.625 | 0.390 | -0.118 | **72** | 0.391 | 0.153 | -0.415 | **97** | 0.307 | 0.094 | -0.761 |
| **23** | 0.392 | 0.153 | -0.412 | **48** | 0.507 | 0.257 | -0.298 | **73** | 0.530 | 0.281 | -0.175 | **98** | 0.257 | 0.066 | -0.460 |
| **24** | 0.471 | 0.222 | -0.141 | **49** | 0.308 | 0.095 | -0.681 | **74** | 0.487 | 0.237 | -0.185 | **99** | 0.292 | 0.085 | -0.510 |
| **25** | 0.365 | 0.133 | -0.526 | **50** | 0.526 | 0.276 | -0.284 | **75** | 0.575 | 0.330 | -0.099 | **100** | 0.417 | 0.174 | -0.477 |

| **Random Models Parameters** | **Average R_Rand_** | **Average R^2^_Rand_** | **Average Q^2^_cv (Rand)_** | **cRp^2^** |
| --- | --- | --- | --- | --- |
|  | 0.438 | 0.208 | -0.331 | 0.619 |

**Table S4.** Calculated anticancer activity and leverages (h) for the new designed 1,2,4-triazin-3(2H)-ones derivatives

|  | | | | | | | | | | | | | | | | |
| --- | --- | --- | --- | --- | --- | --- | --- | --- | --- | --- | --- | --- | --- | --- | --- | --- |
| **N-Pred** | **R1** | **R2** | **R3** | | **R4** | **R5** | **R6** | **R7** | **R8** | **R9** | **R10** | **R11** | | **pIC50** | **hi** | **Com-ment** |
| **1** | H | H | **F** | | H | H | H | **OCH_3_** | H | **OCH_3_** | H | **OH** | | 5.56 | 0.19 | Inside |
| **2** | H | H | **Cl** | | H | H | H | **OCH_3_** | H | **OCH_3_** | H | **OH** | | 6.95 | 0.27 | Inside |
| **3** | H | H | **F** | | H | H | H | **OCH_3_** | H | **F** | H | **OH** | | 6.09 | 0.21 | Inside |
| **4** | H | H | **OCH_3_** | | H | H | H | **F** | H | **F** | H | **OH** | | 5.43 | 0.19 | Inside |
| **5** | H | H | **F** | | H | H | H | **F** | H | **F** | H | **OH** | | 6.62 | 0.24 | Inside |
| **6** | H | H | **OCH_3_** | | H | H | H | **OCH_3_** | H | **Br** | H | **OH** | | 9.91 | 5.64 | Outlier |
| **7** | H | H | **F** | | H | H | H | **OH** | H | **OH** | H | **OH** | | 8.21 | 3.15 | Outlier |
| **8** | H | H | **F** | | H | H | **F** | **OH** | H | **OH** | **F** | **OH** | | 9.24 | 3.89 | Outlier |
| **9** | H | H | **Cl** | | H | H | H | H | H | H | H | **OH** | | 6.62 | 0.18 | Inside |
| **10** | **F** | H | **OCH_3_** | | H | H | H | H | H | H | H | **OH** | | 4.81 | 0.11 | Inside |
| **11** | H | H | **OCH_3_** | | H | H | H | H | H | H | H | **COOH** | | 5.54 | 0.52 | Inside |
| **12** | H | H | **OCH_3_** | | **F** | H | H | H | **OCH_3_** | H | H | **COOH** | | 6.08 | 0.29 | Inside |
| **13** | H | **OCH_3_** | **OCH_3_** | | **F** | H | H | H | **OCH_3_** | H | H | **COOH** | | 5.97 | 0.47 | Inside |
| **14** | H | **OCH_3_** | **OCH_3_** | | **F** | H | H | H | **OCH_3_** | H | H | **OH** | | 4.86 | 0.13 | Inside |
| **15** | H | H | H | | H | **F** | H | H | **OCH_3_** | H | H | **OH** | | 4.79 | 0.11 | Inside |
| **16** | H | **Cl** | **OCH_3_** | | H | **OH** | H | H | H | H | H | **OH** | | 6.43 | 3.12 | Outlier |
| **17** | H | H | **OCH_3_** | | H | H | H | **OCH_3_** | H | **OCH_3_** | **Cl** | **OH** | | 6.48 | 0.77 | Outlier |
| **18** | H | H | **OCH_3_** | | H | H | H | **OCH_3_** | H | **OCH_3_** | **NH_2_** | **OH** | | 5.61 | 1.88 | Outlier |
| **19** | **NH_2_** | H | **OCH_3_** | | H | H | H | **OCH_3_** | H | **OCH_3_** | H | **OH** | | 5.93 | 1.61 | Outlier |
| **20** | H | **NH_2_** | **OCH_3_** | | H | H | H | **OCH_3_** | H | **OCH_3_** | H | **OH** | | 5.91 | 1.47 | Outlier |
| **21** | H | H | **OCH_3_** | | H | H | H | **NH_2_** | H | **NH_2_** | H | **OH** | | 6.44 | 3.18 | Outlier |
| **22** | H | H | **NH_2_** | | H | H | H | **NH_2_** | H | **NH_2_** | H | **OH** | | 7.74 | 7.60 | Outlier |
| **23** | H | H | H | | H | H | H | **NH_2_** | **NH_2_** | **NH_2_** | H | **OH** | | 7.96 | 6.58 | Outlier |
| **24** | H | H | H | | H | H | H | **NH_2_** | **NH_2_** | **NH_2_** | **NH_2_** | **OH** | | 8.73 | 10 | Outlier |
| **25** | H | H | H | | H | H | **NH_2_** | **NH_2_** | **NH_2_** | **NH_2_** | **NH_2_** | **OH** | | 9.68 | 14 | Outlier |
| **26** | H | H | **F** | | **NH_2_** | H | H | **OCH_3_** | H | **OCH_3_** | H | **OH** | | 6.61 | 1.35 | Outlier |
| **27** | **CH_3_** | H | **Cl** | | H | H | H | H | H | H | H | **OH** | | 7.10 | 0.36 | Inside |
| **28** | H | **CH_3_** | **OCH_3_** | | **F** | H | H | H | **OCH_3_** | H | H | **COOH** | | 6.38 | 0.20 | Inside |
| **Table S5.** Physico-Chemical radar chart of the selected lead compounds in the dataset | | | | | | | | | | | | | | | | |
| **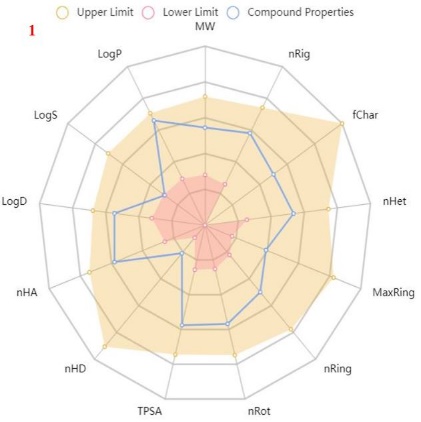** | | | | **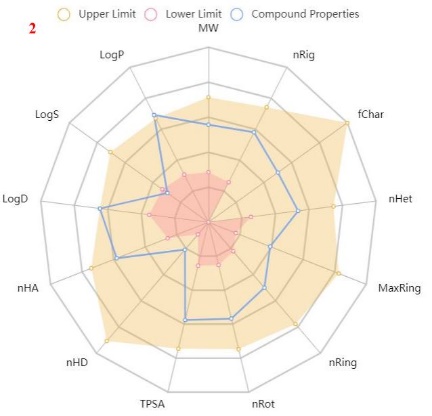** | | | | | **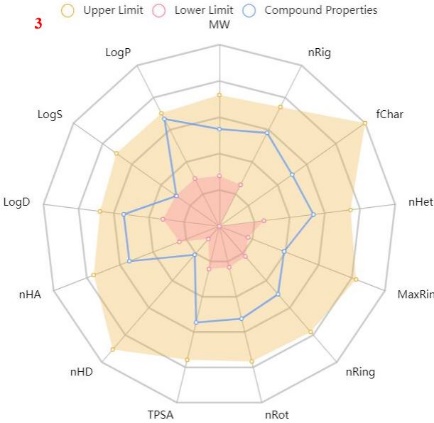** | | | | **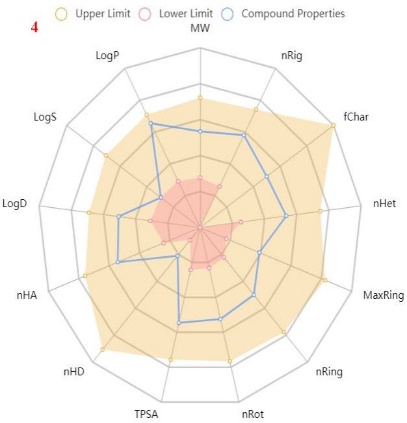** | | | |
| **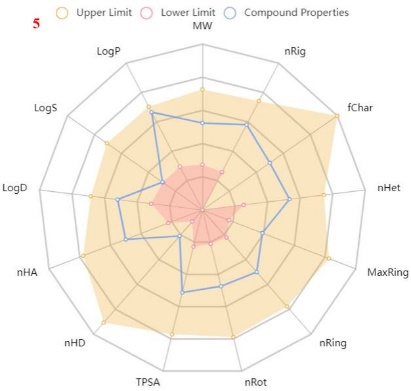** | | | | **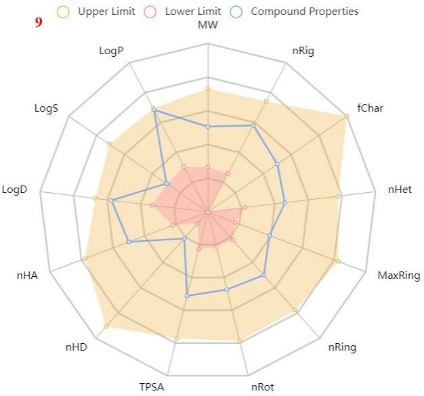** | | | | | **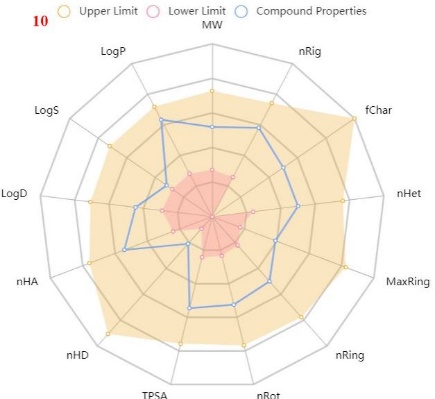** | | | | **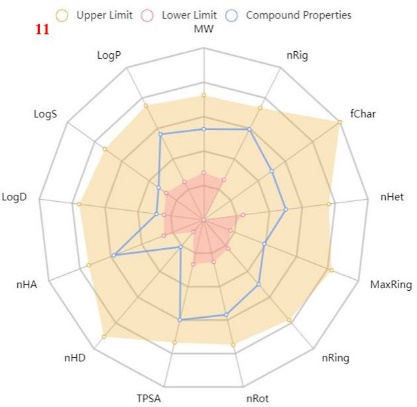** | | | |
| **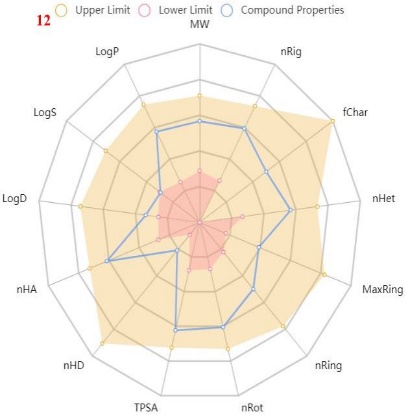** | | | | **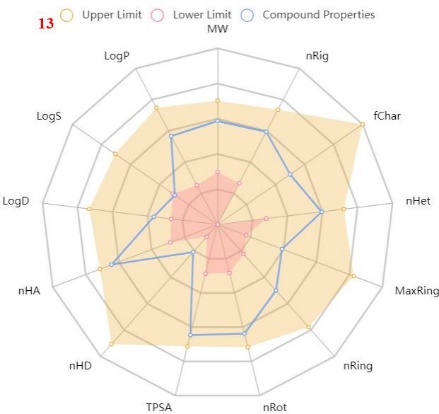** | | | | | **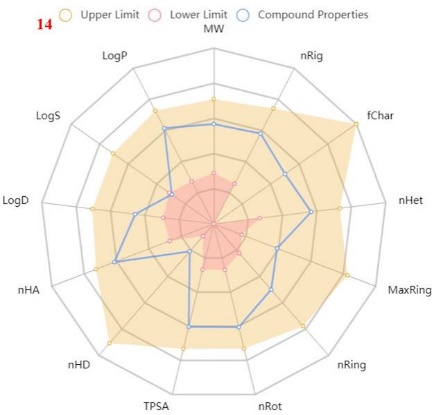** | | | | **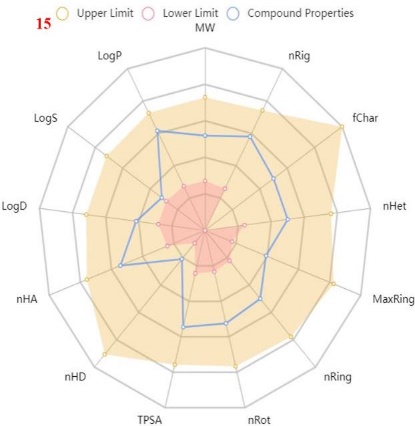** | | | |
| **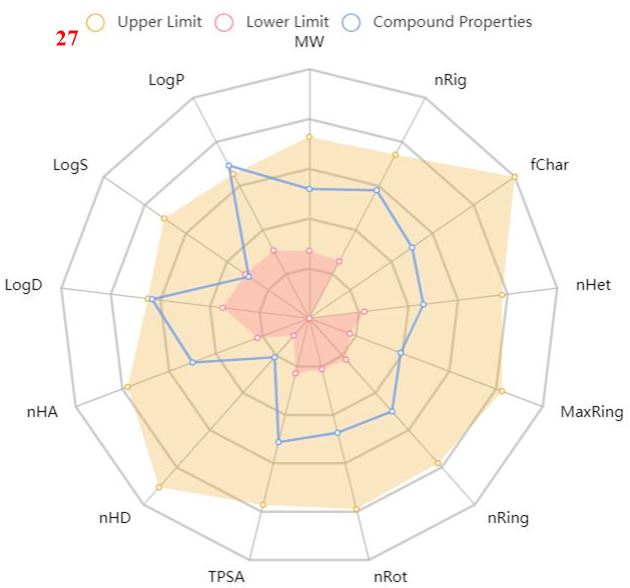** | | | | | | | | | **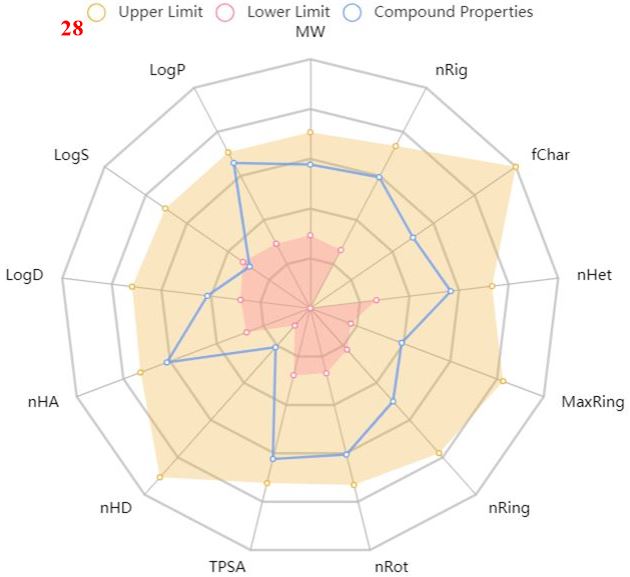** | | | | | | | |

**Table S6.** Lipinski-Veber-Egan properties of the new designed 1,2,4-triazin-3(2H)-ones derivatives

| **Inhibitor** | **Property** | | | | | | | | |
| --- | --- | --- | --- | --- | --- | --- | --- | --- | --- |
|  | **MW** | **LogP** | **NROT** | **NHA** | **NHD** | **TPSA** | **Lipinski’s violations** | **Veber violations** | **Egan violations** |
| **Rule** | **<500** | **<=5** | **<10** | **<10** | **<5** | **<140** | **<=1** | **<=1** | **<=1** |
| Pred1 | 401.39 | 2.64 | 7 | 6 | 1 | 91.67 | 0 | 0 | 0 |
| Pred2 | 417.84 | 3.1 | 7 | 5 | 1 | 91.67 | 0 | 0 | 0 |
| Pred3 | 389.35 | 2.9 | 6 | 6 | 1 | 82.44 | 0 | 0 | 0 |
| Pred4 | 389.35 | 2.9 | 6 | 6 | 1 | 82.44 | 0 | 0 | 0 |
| Pred5 | 377.32 | 3.15 | 5 | 6 | 1 | 73.21 | 0 | 0 | 0 |
| Pred9 | 357.79 | 3.29 | 5 | 3 | 1 | 73.21 | 0 | 0 | 0 |
| Pred10 | 371.36 | 2.73 | 6 | 5 | 1 | 82.44 | 0 | 0 | 0 |
| Pred11 | 381.38 | 2.22 | 7 | 5 | 1 | 99.51 | 0 | 0 | 0 |
| Pred12 | 429.40 | 2.28 | 8 | 7 | 1 | 108.74 | 0 | 0 | 0 |
| Pred13 | 459.43 | 1.93 | 9 | 8 | 1 | 117.97 | 0 | 0 | 0 |
| Pred14 | 431.15 | 2.28 | 8 | 7 | 1 | 100.9 | 0 | 0 | 0 |
| Pred15 | 371.13 | 2.73 | 6 | 5 | 1 | 82.44 | 0 | 0 | 0 |
| Pred27 | 371.1 | 3.73 | 5 | 3 | 1 | 73.21 | 0 | 0 | 0 |
| Pred28 | 443.15 | 2.51 | 8 | 7 | 1 | 108.74 | 0 | 0 | 0 |

| **Table S7.** ADMET/pharmacokinetic features of the best-hit compounds | | | | | | | | | | | | | | | | | |
| --- | --- | --- | --- | --- | --- | --- | --- | --- | --- | --- | --- | --- | --- | --- | --- | --- | --- |
| **Property** | | | | **ADMET report –Pred-** | | | | | | | | | | | | | |
|  |  |  |  | **1** | **2** | **3** | **4** | **5** | **9** | **10** | **11** | **12** | **13** | **14** | **15** | **27** | **28** |
| **Absorption** | Human intestinal absorption | | | 70.391 | 71.152 | 95.964 | 95.57 | 92.145 | 91.514 | 96.568 | 66.817 | 66.433 | 63.568 | 69.405 | 95.563 | 95.501 | 66.82 |
|  | Skin permeability (logkp) | | | -2.703 | -2.708 | -2.702 | -2.692 | -2.741 | -2.652 | -2.68 | -2.735 | -2.735 | -2.735 | -2.712 | -2.695 | -2.666 | -2.735 |
| **Distribution** | VDss (human)  (log L/kg) | | | -1.112 | -1.04 | -1.072 | -1.097 | -0.958 | -0.696 | -1.022 | -0.874 | -0.854 | -0.794 | -1.039 | -1.259 | -0.795 | -0.775 |
|  | BBB permeability  (log BB) | | | -1.136 | -1.103 | -1.127 | -1.148 | -1.166 | -0.701 | -0.913 | -0.908 | -1.314 | -1.544 | -1.361 | -0.895 | -0.652 | -1.312 |
|  | CNS permeability  (log PS) | | | -3.245 | -2.594 | -3.189 | -3.147 | -3.189 | -2.278 | -2.617 | -2.814 | -3.412 | -3.543 | -3.353 | -3.353 | -2.179 | -3.351 |
| **Metabolism** | Cytochrome P450  (CYP450) | Substrate | 2D6 | No | No | No | Yes | No | No | No | No | No | No | No | No | Yes | No |
|  |  |  | 3A4 | Yes | Yes | Yes | Yes | Yes | Yes | Yes | Yes | Yes | Yes | Yes | Yes | Yes | Yes |
|  |  | Inhibitor | 1A2 | No | No | No | No | No | No | No | No | No | No | No | No | No | No |
|  |  |  | 2C19 | No | No | No | No | No | No | No | No | No | No | No | No | No | No |
|  |  |  | 2C9 | No | No | No | No | No | No | No | No | No | No | No | No | No | No |
|  |  |  | 2D6 | No | No | No | No | No | No | No | No | No | No | No | No | No | No |
|  |  |  | 3A4 | No | No | No | No | No | No | No | No | No | No | No | No | No | No |
| **Excretion** | Total Clearance  (log ml/min/k) | | | 0.323 | 0.902 | 0.134 | 0.486 | -0.042 | 0.402 | 0.39 | 0.446 | 0.634 | 0.695 | 0.612 | 0.267 | 0.367 | 0.631 |
| **Toxicity** | AMES toxicity | | | No | No | No | No | No | No | No | No | No | No | No | No | No | No |
|  | Skin Sensitisation | | | No | No | No | No | No | No | No | No | No | No | No | No | No | No |
|  | Hepatotoxicity | | | Yes | Yes | Yes | Yes | No | No | Yes | No | Yes | No | Yes | Yes | No | No |
|  | Carcinogenicity | | | No | No | No | No | No | No | No | Yes | No | No | No | No | No | No |
|  | Mutagenicity | | | No | No | No | No | No | No | No | No | No | No | No | No | No | No |

|  | | |
| --- | --- | --- |
| **Cp** | **3D representations of the binding interactions** | **2D representations of the binding interactions** |
| **Ligand**  **Pred1 –** **Tubulin** | 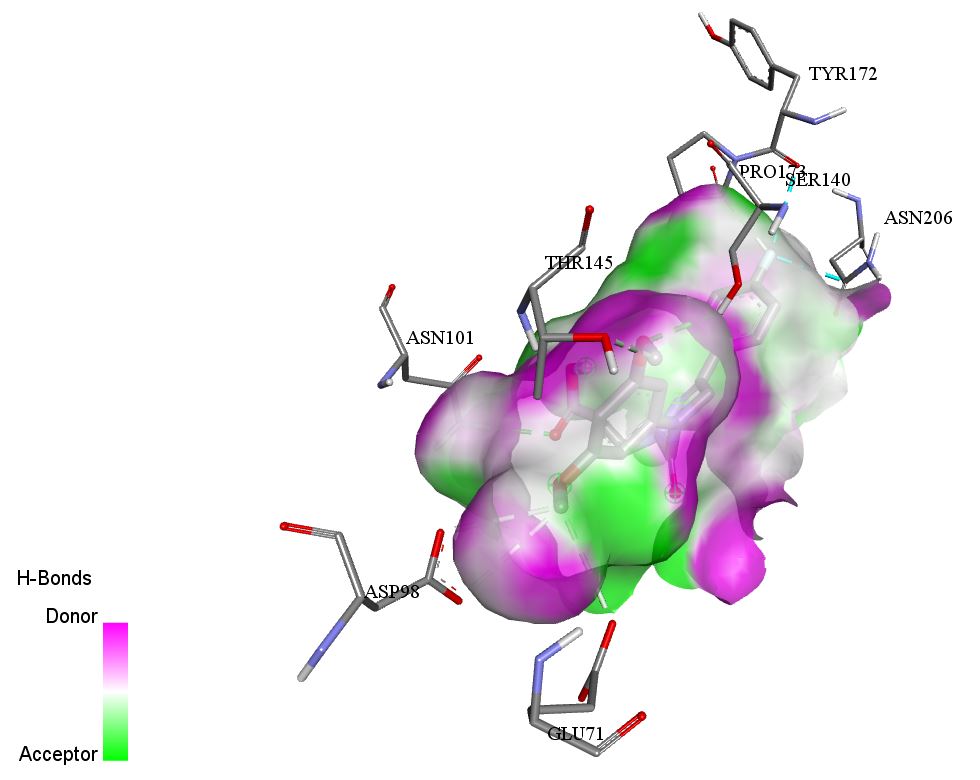 | 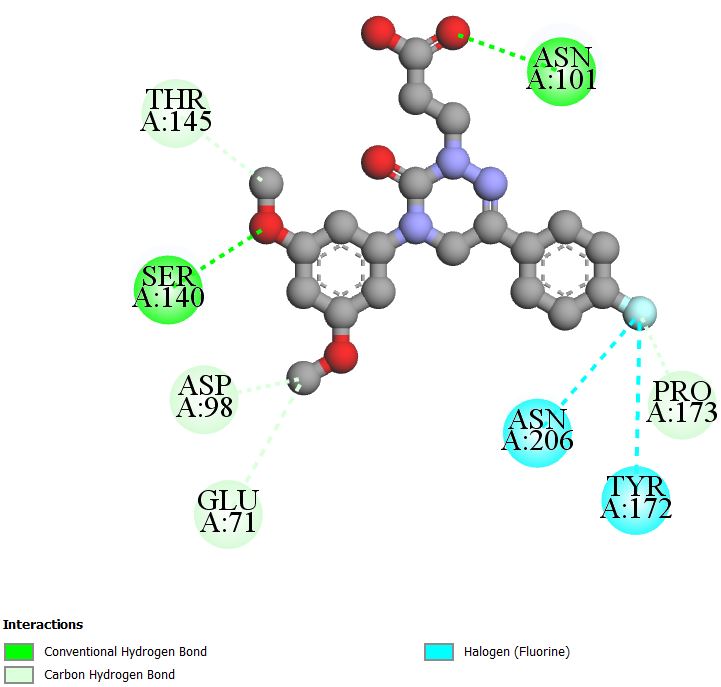 |
| **Ligand**  **Pred2–** **Tubulin** | 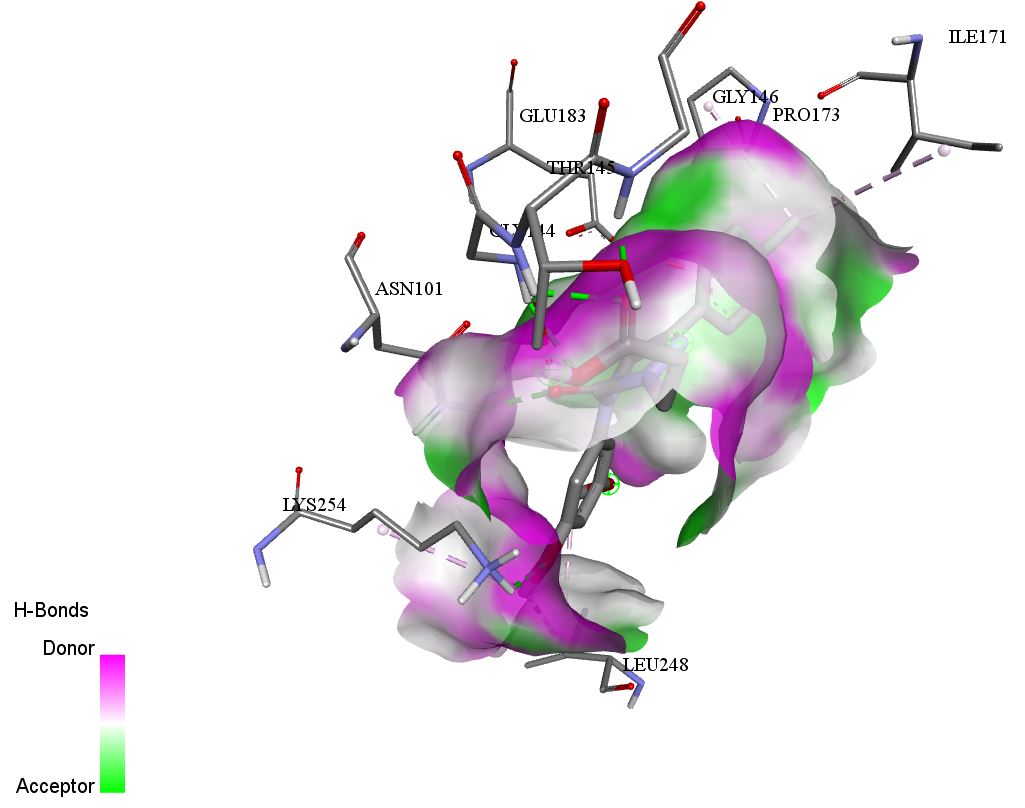 | 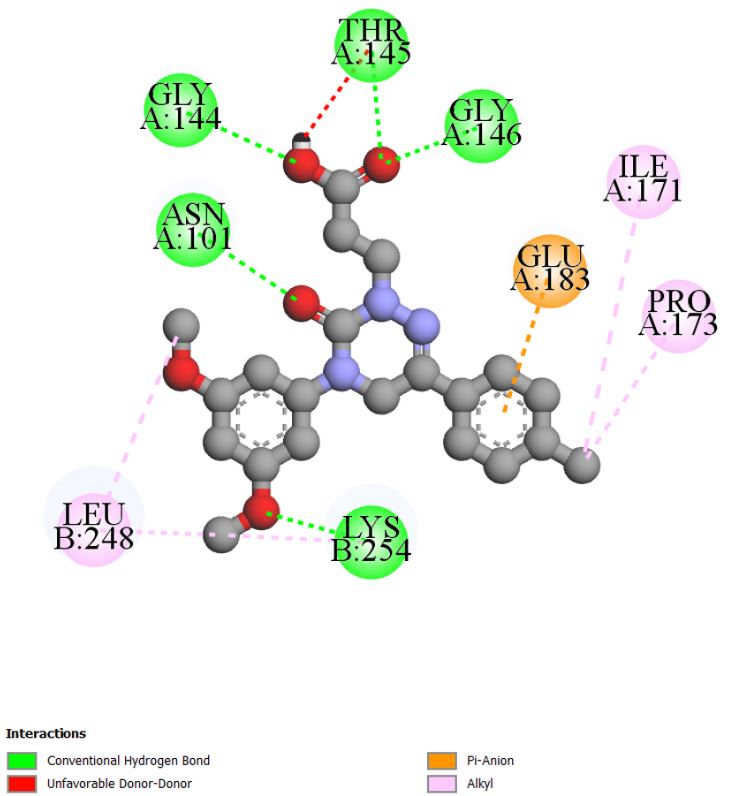 |
| **Ligand**  **Pred3 –** **Tubulin** | 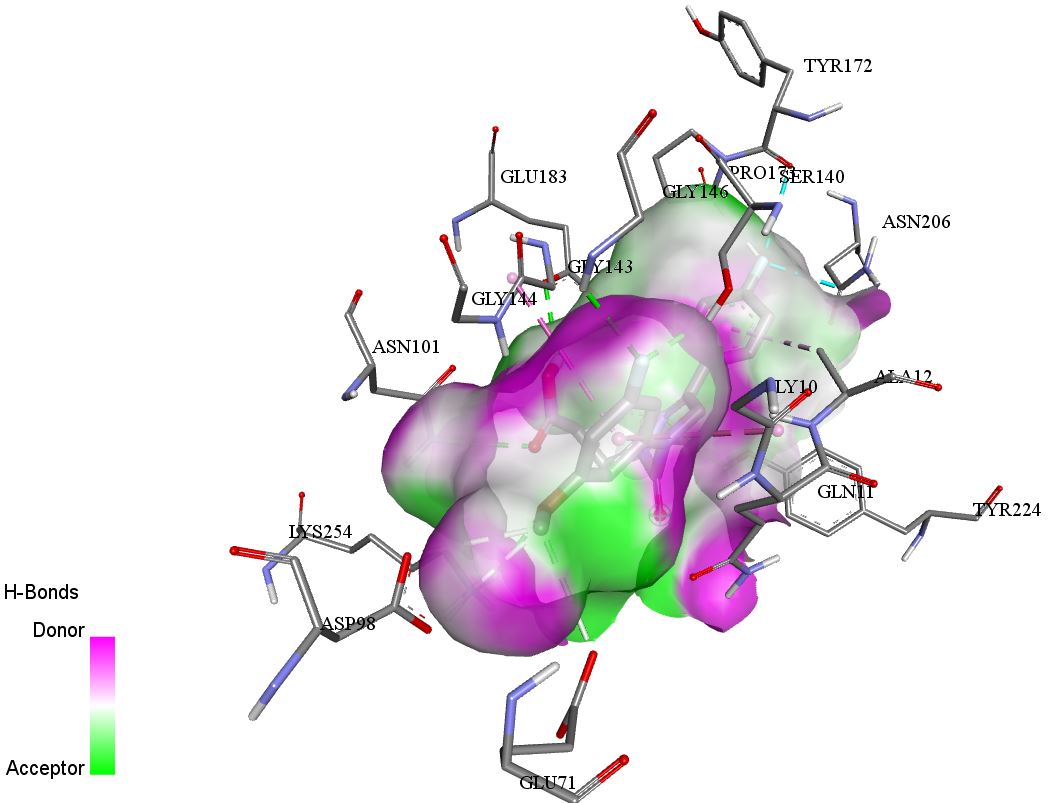 | 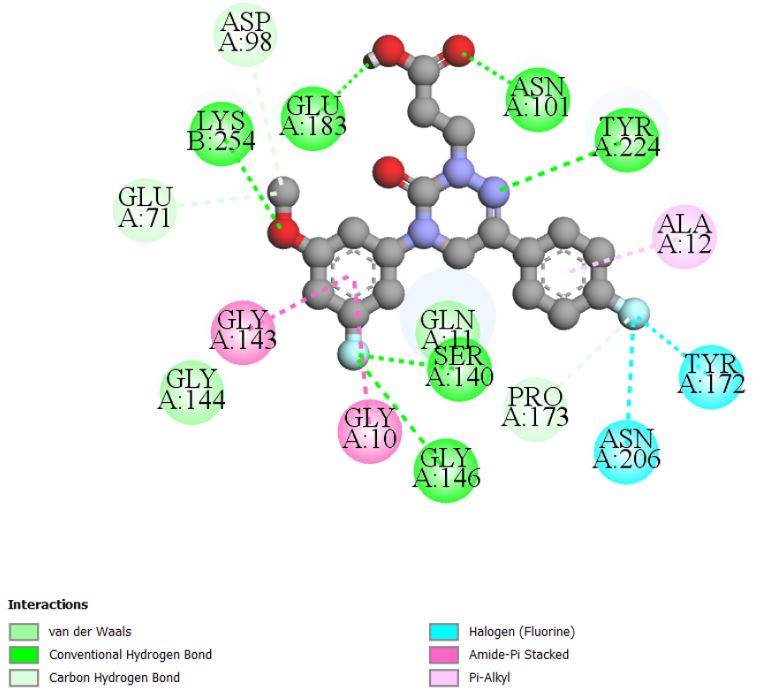 |
| **Ligand**  **Pred4 –** **Tubulin** | 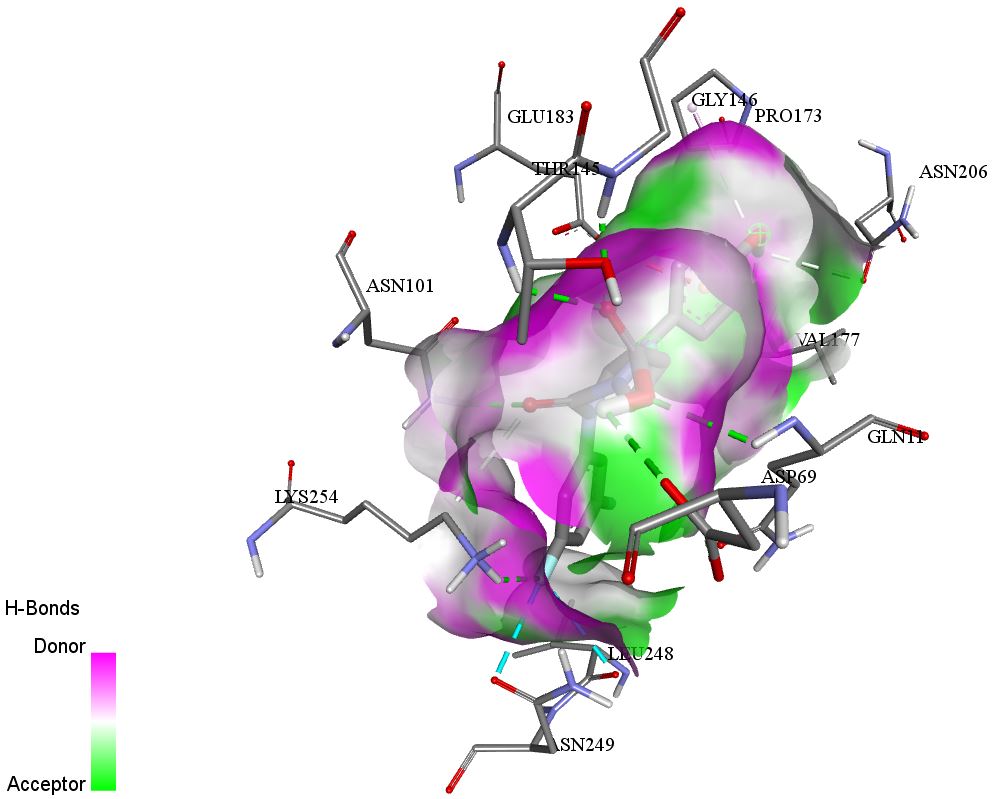 | 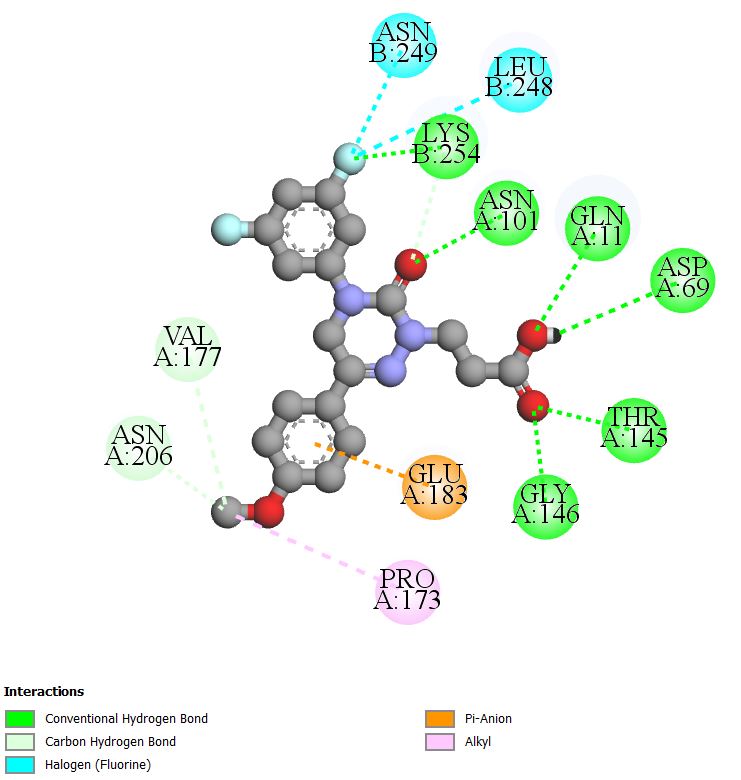 |
| **Ligand**  **Pred5 –** **Tubulin** | 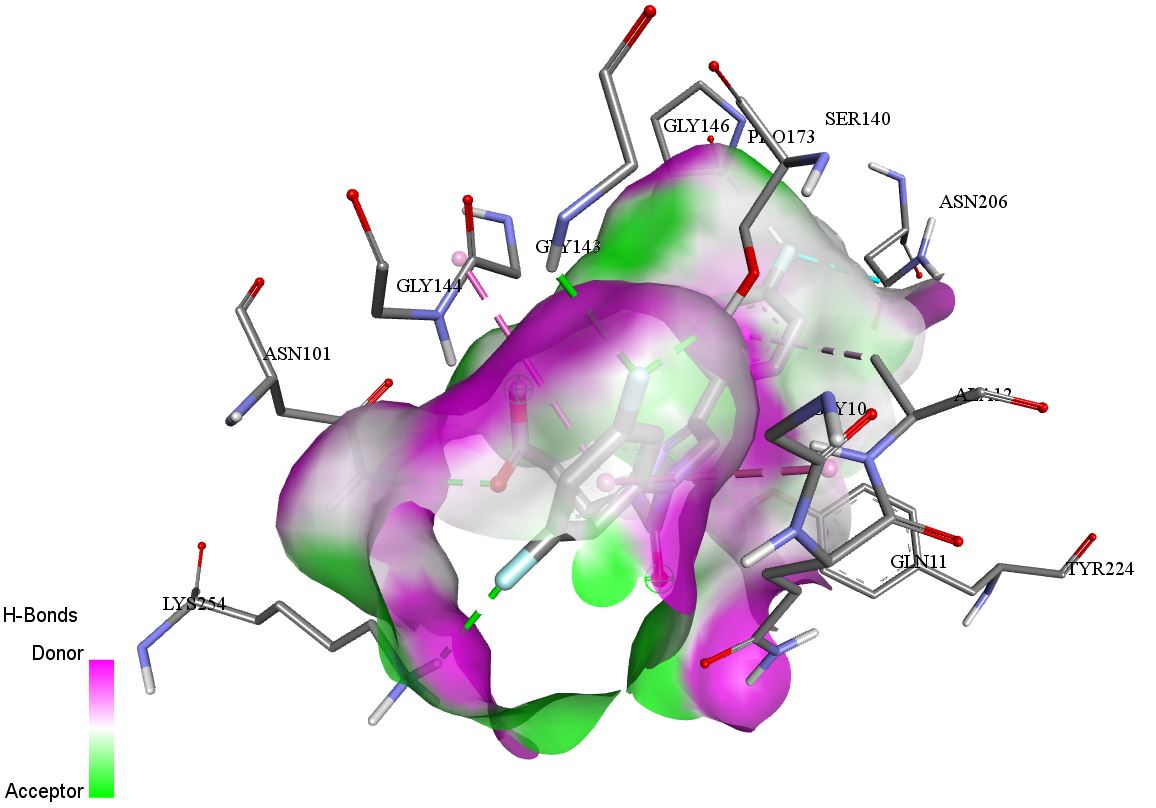 | 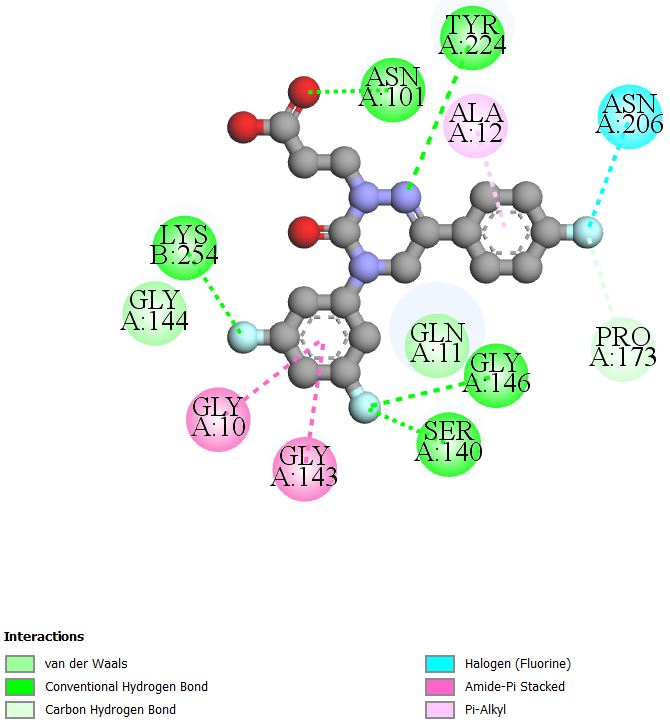 |
| **Ligand**  **Pred9 –** **Tubulin** | 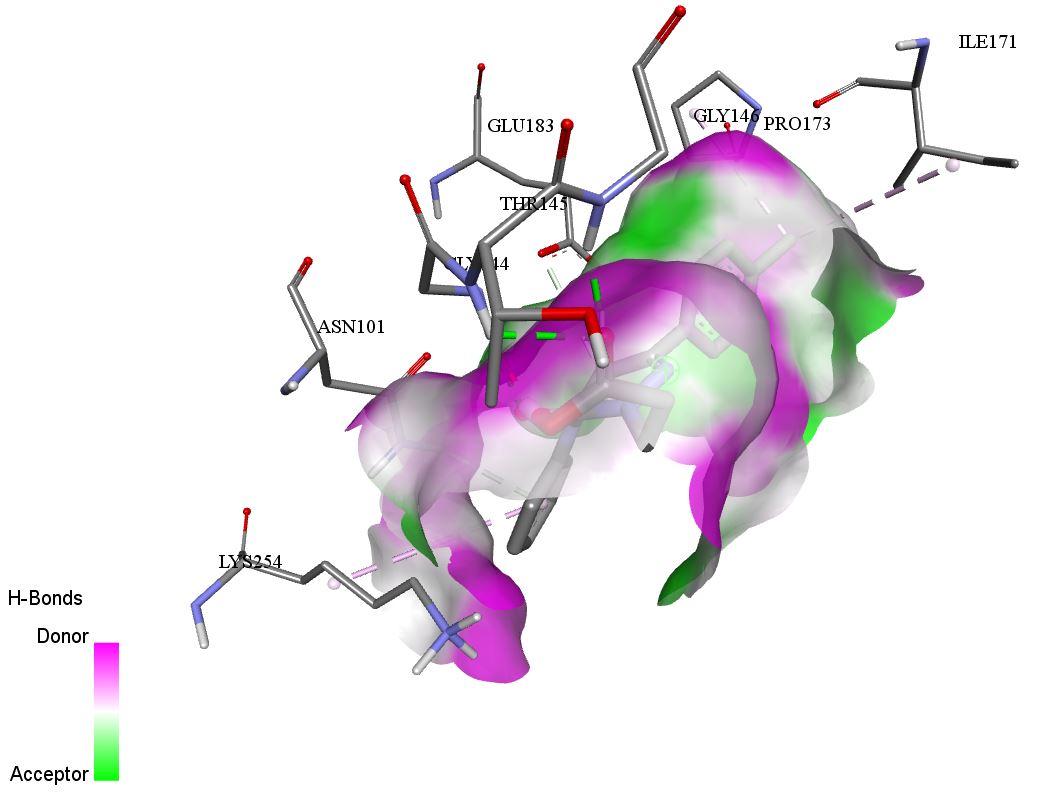 | 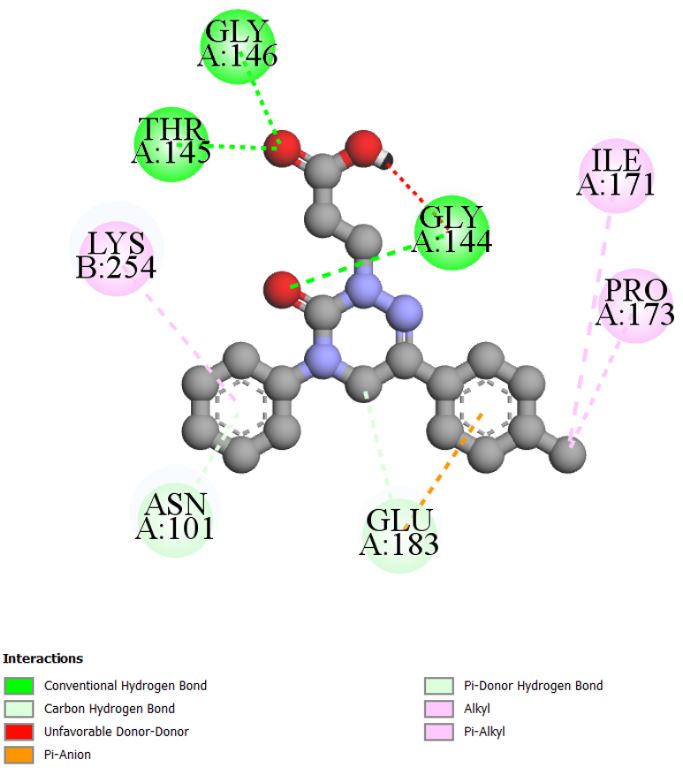 |
| **Ligand**  **Pred10 –** **Tubulin** | 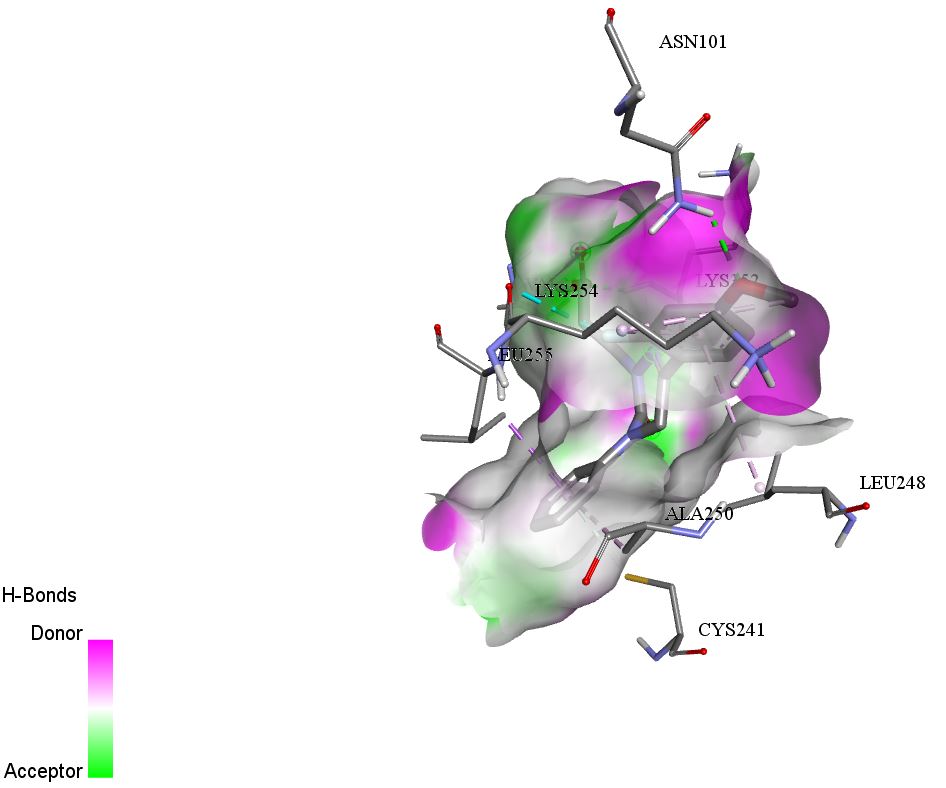 | 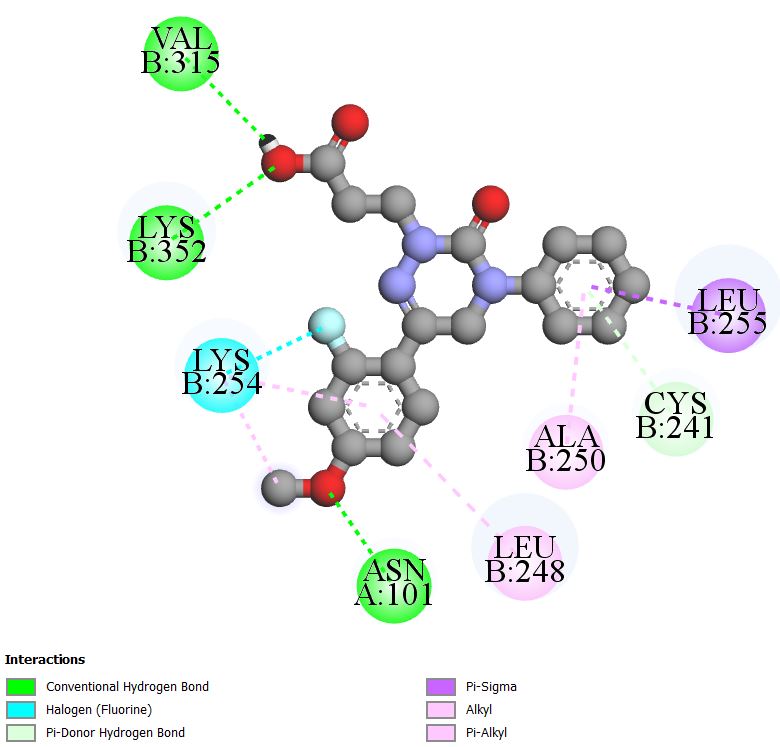 |
| **Ligand**  **Pred11 –** **Tubulin** | 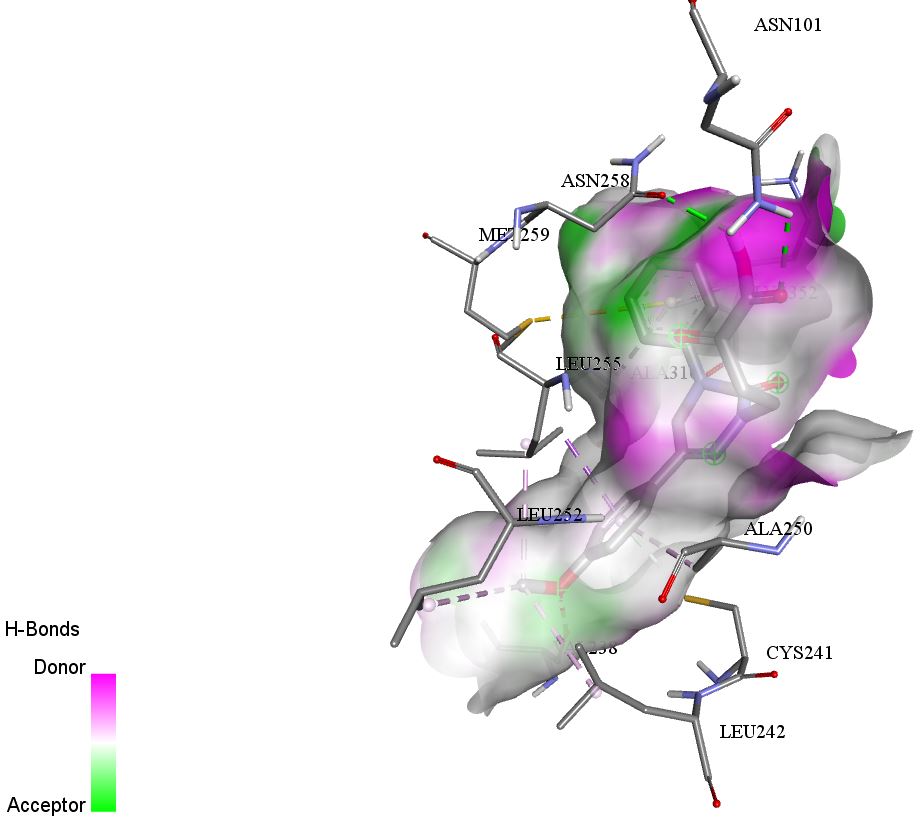 | 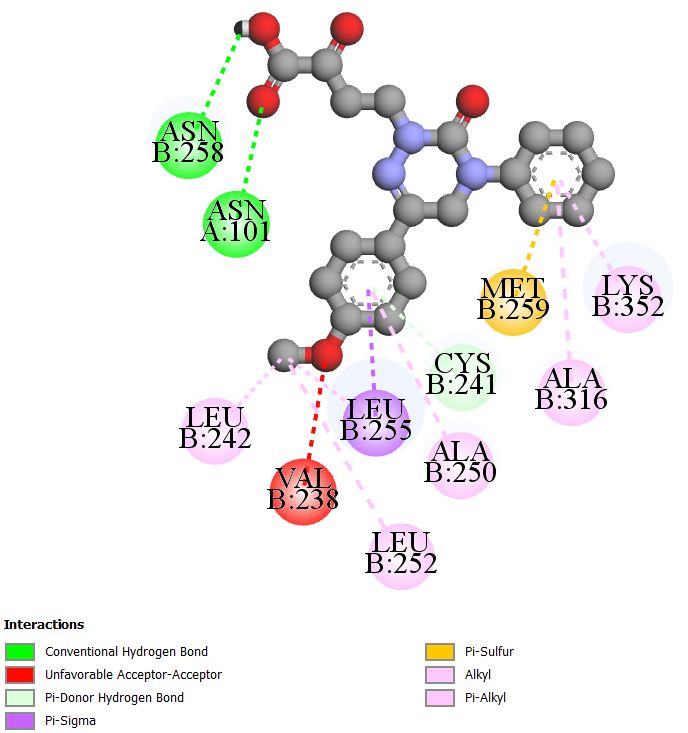 |
| **Ligand**  **Pred12 –** **Tubulin** | 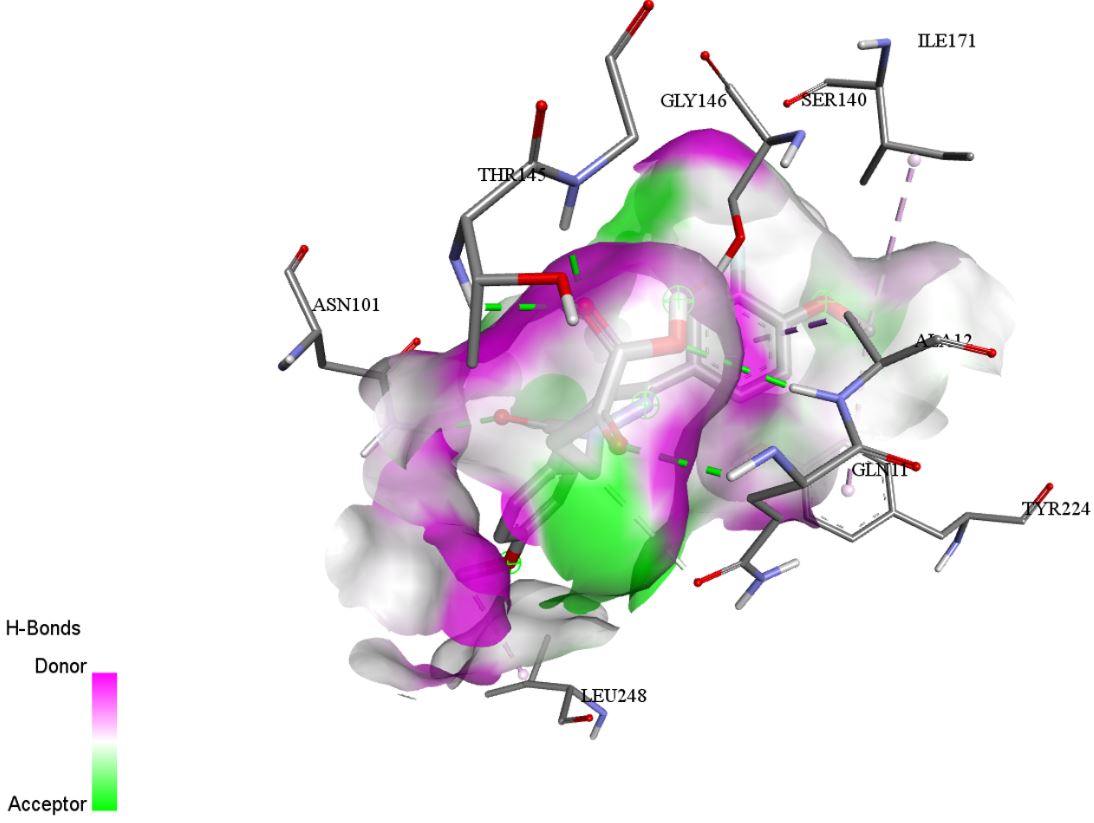 | 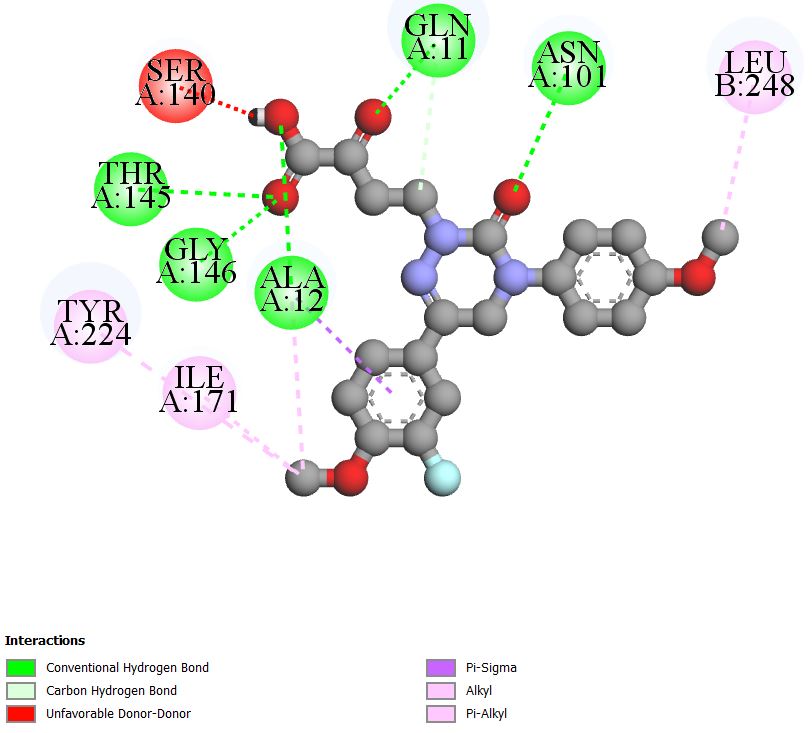 |
| **Ligand**  **Pred13 –** **Tubulin** | 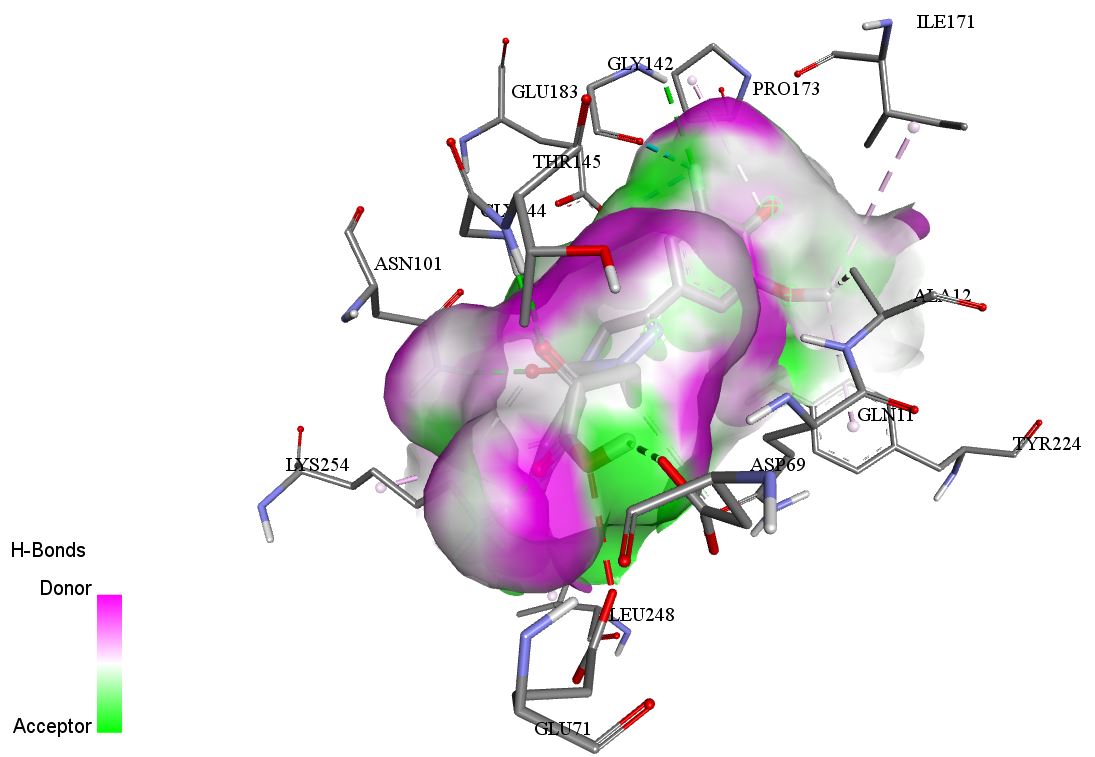 | 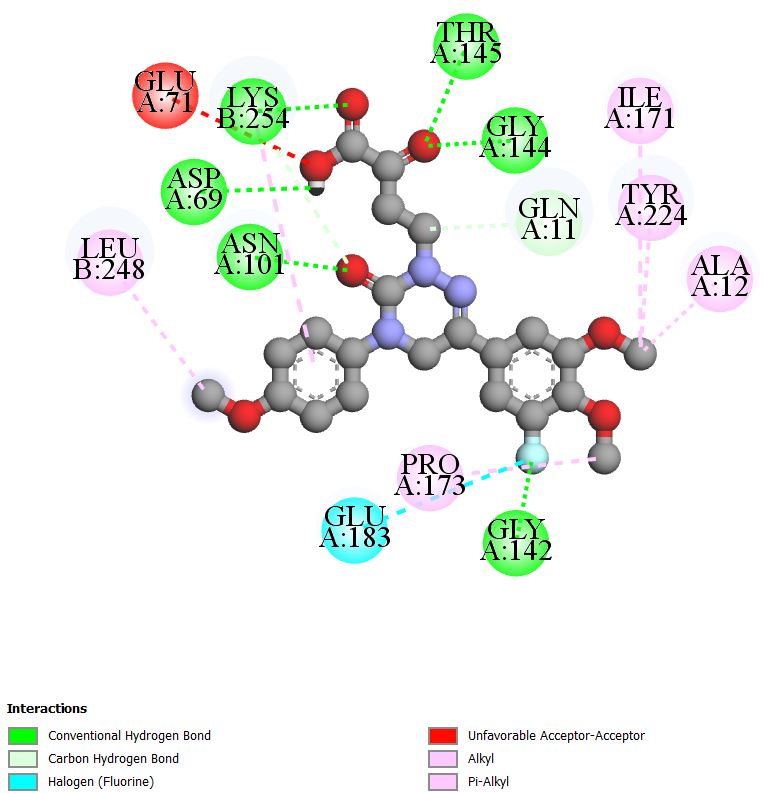 |
| **Ligand**  **Pred14 –** **Tubulin** | 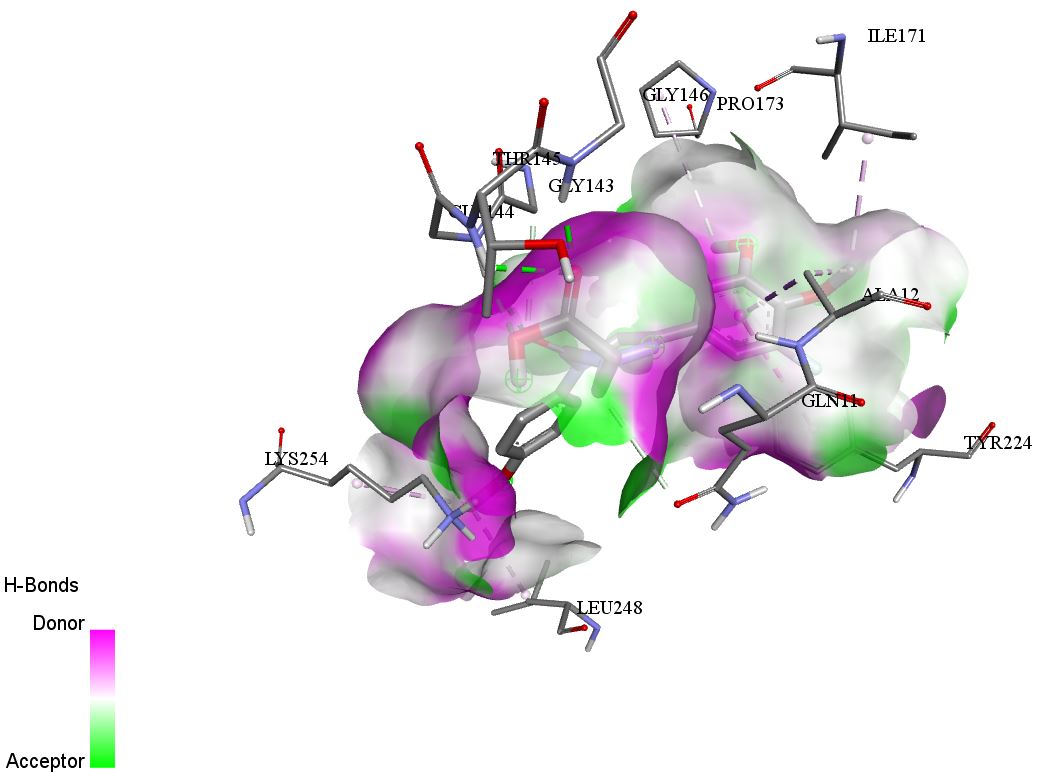 | 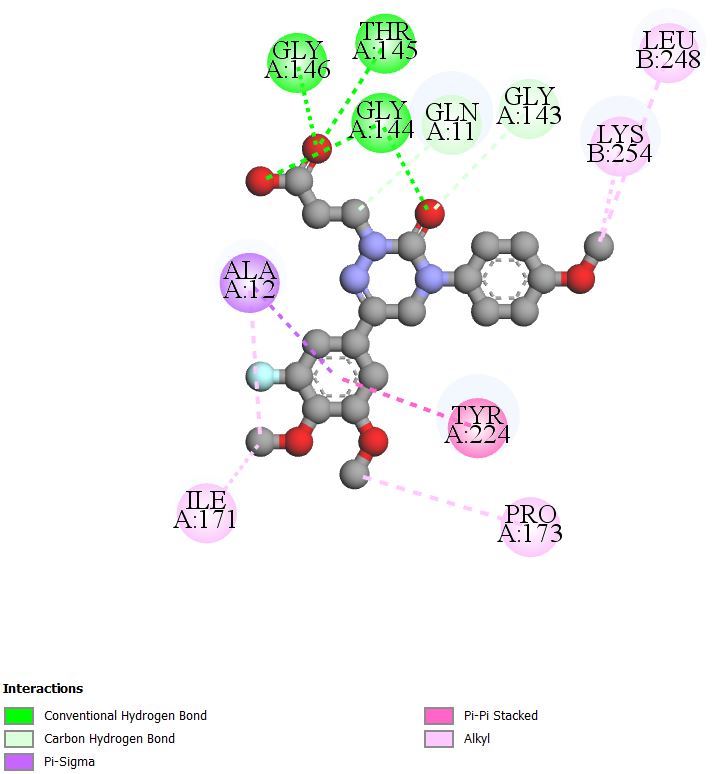 |
| **Ligand**  **Pred15 –** **Tubulin** | 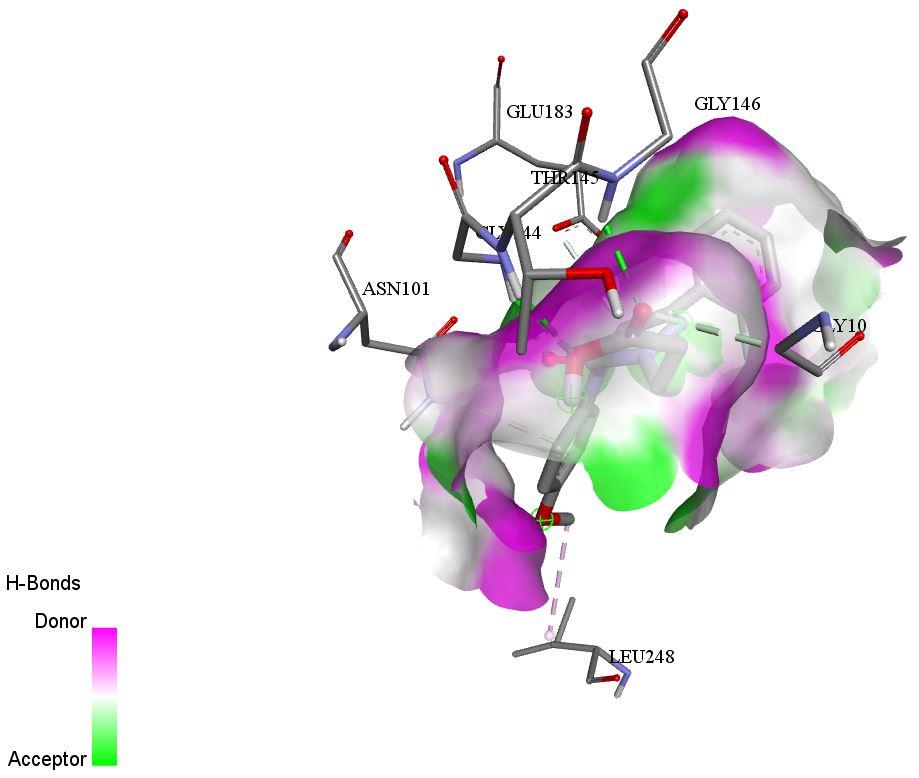 | 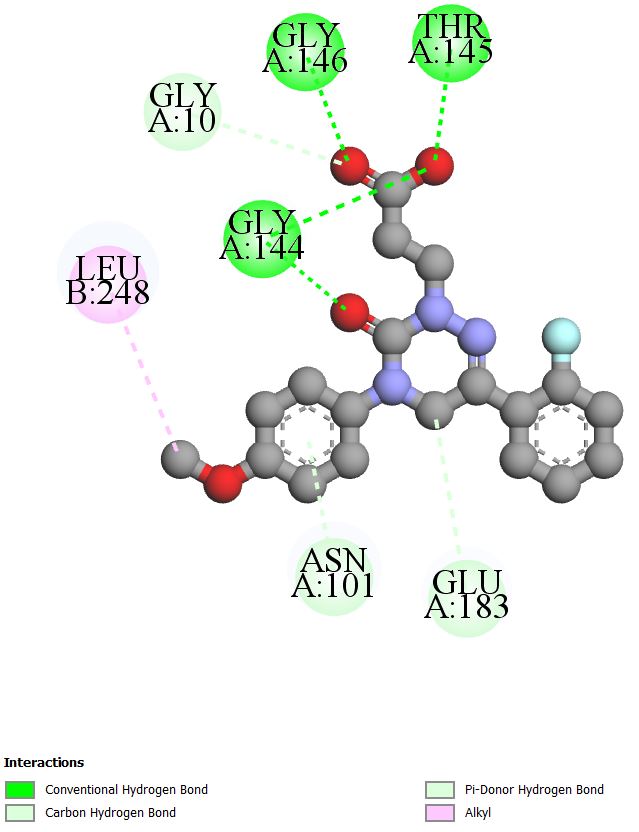 |
| **Ligand**  **Pred27 –** **Tubulin** | 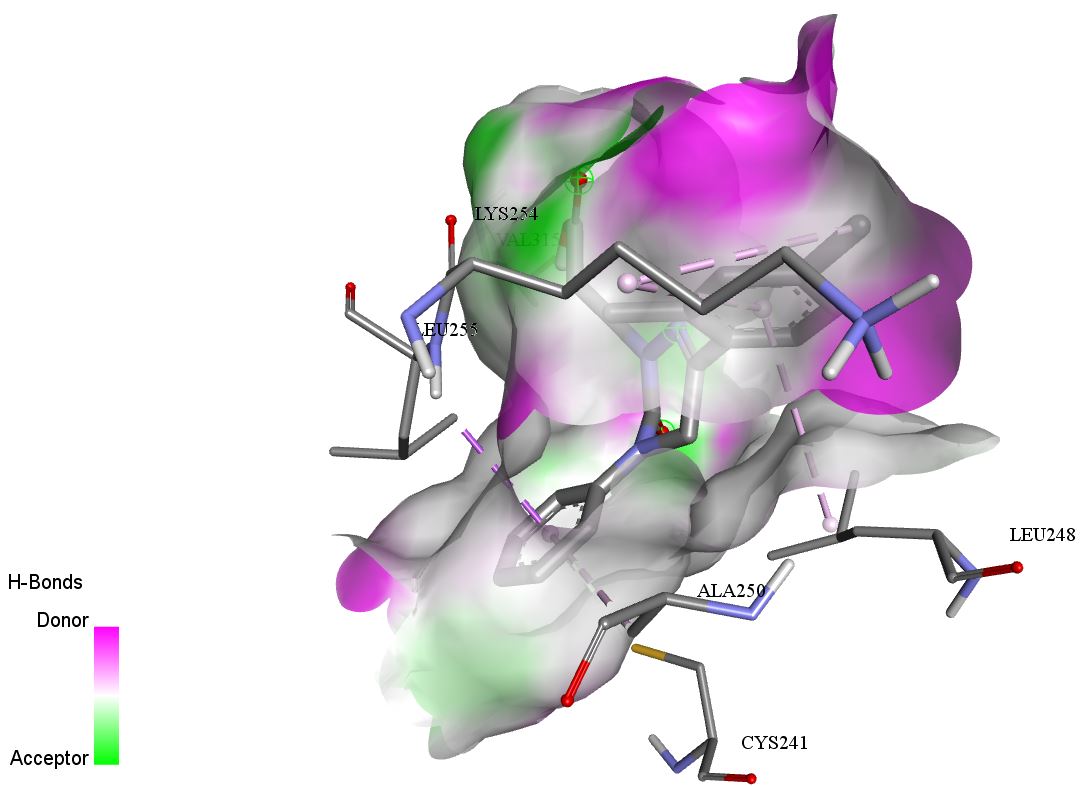 | 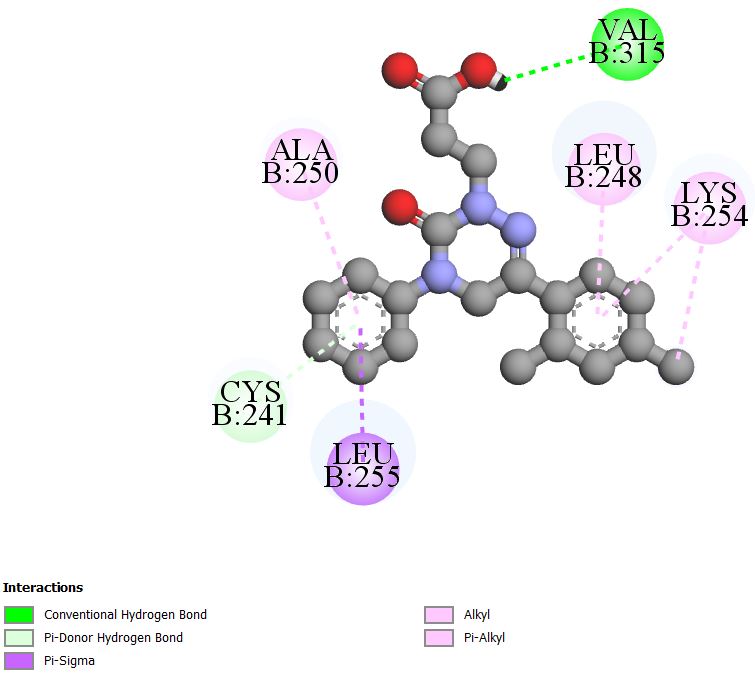 |
| **Ligand**  **Pred28 –** **Tubulin** | 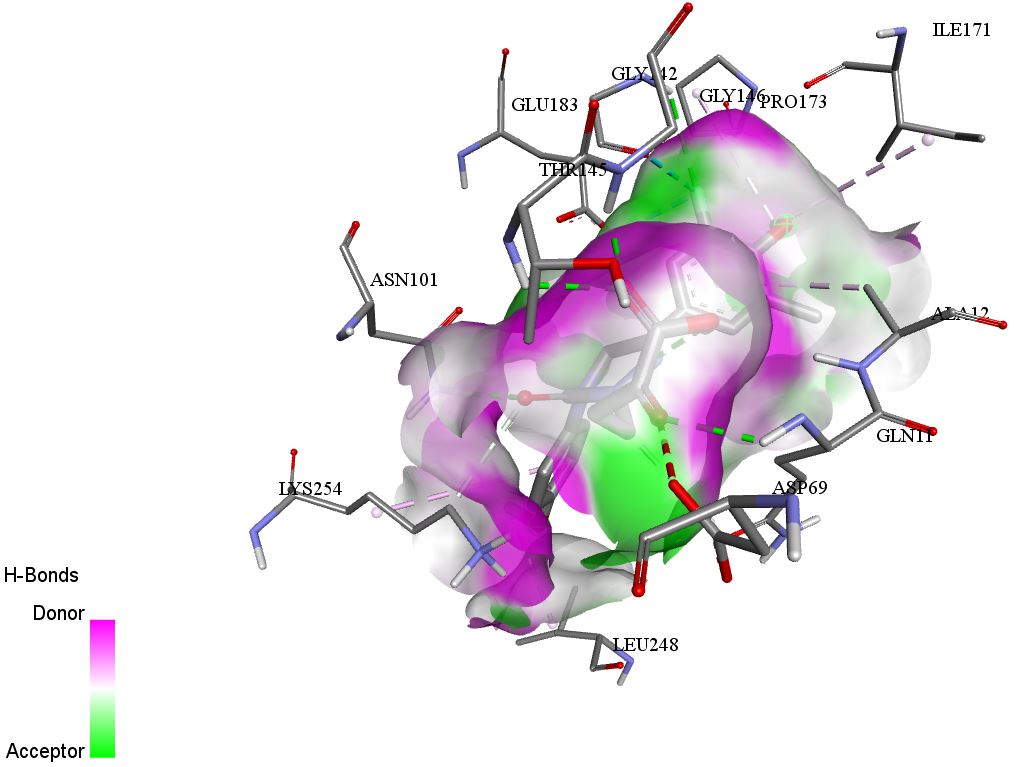 | 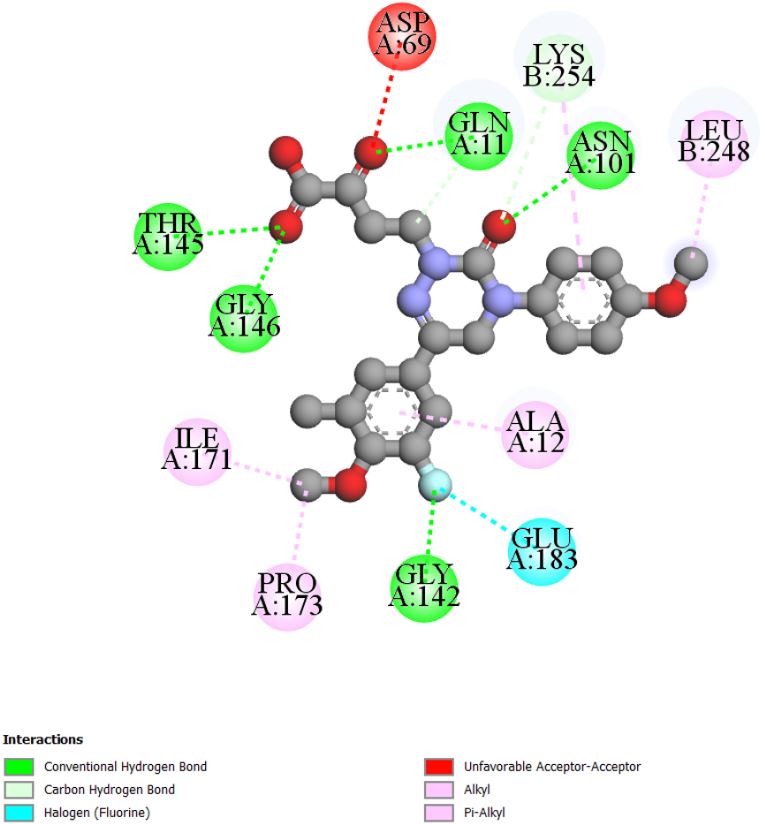 |
| **Co-crystallized ligand –** **Tubulin** | 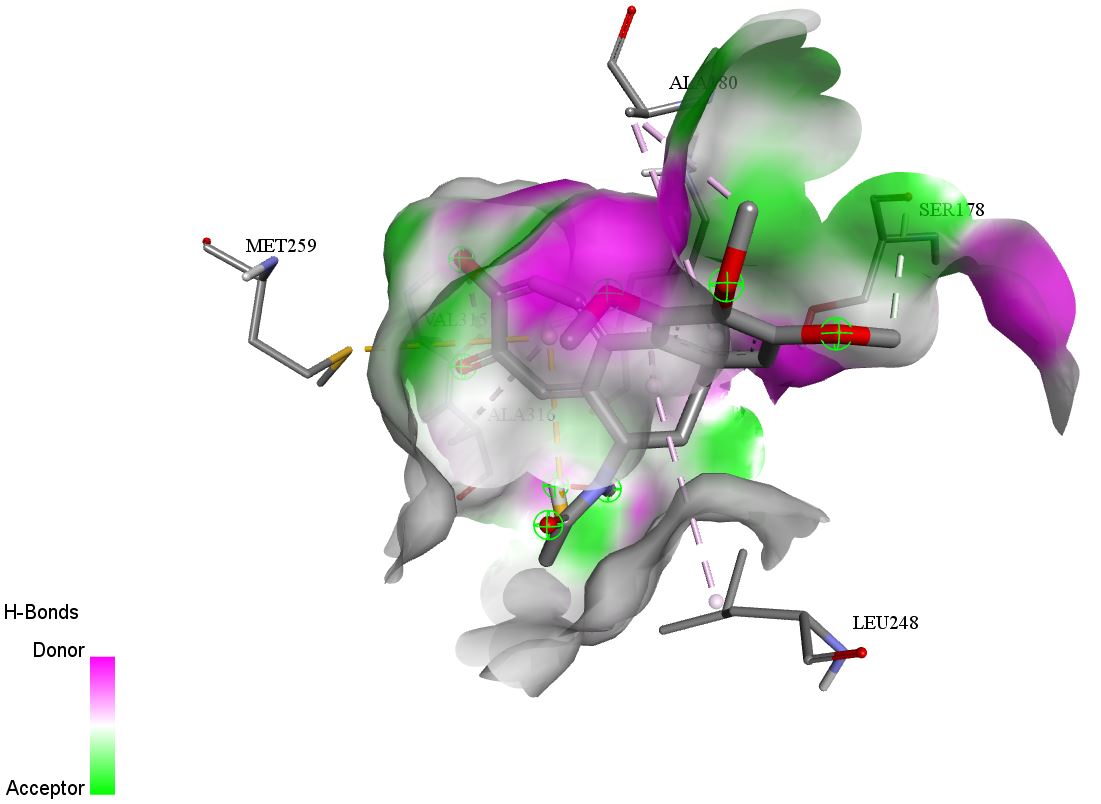 | 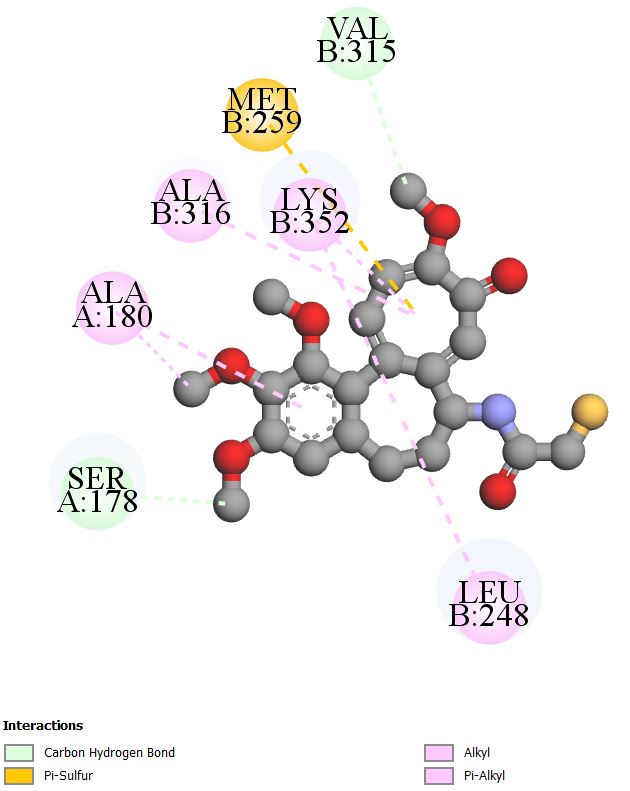 |
| **Fig S1.** 3D and 2D representations of the binding interactions of the 15 complex compounds | | |

**Table S8.** Interaction table between the compounds and 1SA0 protein cancer

| Interactions | **Amino acid** | **Position** | **Distance (Å)** | **Interaction type** |
| --- | --- | --- | --- | --- |
| **Co-crystallized**  **ligand-** **Tubulin** | Ser | A: 178 | 3.52 | Carbon Hydrogen Bond |
|  | Val | B: 315 | 3.59 | Carbon Hydrogen Bond |
|  | Met | B: 259 | 5.63 | Pi-Sulfur |
|  | Ala | B: 316 | 5.45 | Pi-Alkyl |
|  | Ala | A: 180 | 5.10 | Pi-Alkyl |
|  | Lys | B: 352 | 3.85 | Pi-Alkyl |
|  | Leu | B: 248 | 5.25 | Alkyl |
|  | Ala | A: 180 | 3.59 | Alkyl |
|  | Lys | B: 352 | 4.10 | Alkyl |
| **Ligand Pred1 –** **Tubulin** | Ser | A: 140 | 2.59 | Conventional Hydrogen Bond |
|  | Asn | A: 101 | 2.15 | Conventional Hydrogen Bond |
|  | Pro | A: 173 | 3.03 | Carbon Hydrogen Bond |
|  | Glu | A: 71 | 3.46 | Carbon Hydrogen Bond |
|  | Asp | A: 98 | 5.47 | Carbon Hydrogen Bond |
|  | Thr | A: 145 | 3.79 | Carbon Hydrogen Bond |
|  | Asn | A: 206 | 3.13 | Halogen |
|  | Tyr | A: 172 | 3.53 | Halogen |
| **Ligand Pred2 –** **Tubulin** | Gly | A: 144 | 2.54 | Conventional Hydrogen Bond |
|  | Asn | A: 101 | 3.06 | Conventional Hydrogen Bond |
|  | Thr | A: 145 | 2.23 | Conventional Hydrogen Bond |
|  | Gly | A: 146 | 1.92 | Conventional Hydrogen Bond |
|  | Lys | B: 254 | 2.57 | Conventional Hydrogen Bond |
|  | Glu | A: 183 | 3.59 | Pi-Anion |
|  | Ile | A: 171 | 5.31 | Alkyl |
|  | Pro | A: 173 | 4.43 | Alkyl |
|  | Leu | B: 248 | 4.85 | Alkyl |
|  | Lys | B: 254 | 4.40 | Alkyl |
| **Ligand Pred3 –** **Tubulin** | Gly | A: 146 | 2.68 | Conventional Hydrogen Bond |
|  | Asn | A: 101 | 2.18 | Conventional Hydrogen Bond |
|  | Tyr | A: 224 | 2.86 | Conventional Hydrogen Bond |
|  | Glu | A: 183 | 2.49 | Conventional Hydrogen Bond |
|  | Lys | B: 254 | 2.31 | Conventional Hydrogen Bond |
|  | Ser | A: 140 | 2.51 | Conventional Hydrogen Bond |
|  | Pro | A: 173 | 3.13 | Carbon Hydrogen Bond |
|  | Glu | A: 71 | 3.35 | Carbon Hydrogen Bond |
|  | Asp | A: 98 | 5.41 | Carbon Hydrogen Bond |
|  | Asn | A: 206 | 3.14 | Halogen |
|  | Tyr | A: 172 | 3.63 | Halogen |
|  | Gly | A: 143 | 5.12 | Amide -Pi Stacked |
|  | Gly | A: 10 | 5.31 | Amide -Pi Stacked |
|  | Ala | A: 12 | 5.46 | Pi-Alkyl |
| **Ligand Pred4 –** **Tubulin** | Gly | A: 146 | 2.13 | Conventional Hydrogen Bond |
|  | Asn | A: 101 | 2.81 | Conventional Hydrogen Bond |
|  | Gln | A: 11 | 2.62 | Conventional Hydrogen Bond |
|  | Asp | A: 69 | 3.09 | Conventional Hydrogen Bond |
|  | Lys | B: 254 | 2.37 | Conventional Hydrogen Bond |
|  | Thr | A: 145 | 2.17 | Conventional Hydrogen Bond |
|  | Val | A: 177 | 3.42 | Carbon Hydrogen Bond |
|  | Asn | A: 206 | 3.48 | Carbon Hydrogen Bond |
|  | Lys | B: 254 | 3.55 | Carbon Hydrogen Bond |
|  | Asn | B: 249 | 2.97 | Halogen |
|  | Leu | B: 248 | 3.61 | Halogen |
|  | Glu | A: 183 | 3.54 | Pi-Anion |
|  | Pro | A: 173 | 4.88 | Alkyl |
| **Ligand Pred5 –** **Tubulin** | Gly | A: 146 | 2.71 | Conventional Hydrogen Bond |
|  | Asn | A: 101 | 2.25 | Conventional Hydrogen Bond |
|  | Tyr | A: 224 | 2.88 | Conventional Hydrogen Bond |
|  | Ser | A: 140 | 2.57 | Conventional Hydrogen Bond |
|  | Lys | B: 254 | 2.33 | Conventional Hydrogen Bond |
|  | Pro | A: 173 | 3.20 | Carbon Hydrogen Bond |
|  | Asn | A: 206 | 3.18 | Halogen |
|  | Gly | A: 143 | 5.20 | Amide -Pi Stacked |
|  | Gly | A: 10 | 5.21 | Amide -Pi Stacked |
|  | Ala | A: 12 | 5.40 | Pi-Alkyl |
| **Ligand Pred9 –** **Tubulin** | Gly | A: 146 | 1.86 | Conventional Hydrogen Bond |
|  | Thr | A: 145 | 2.30 | Conventional Hydrogen Bond |
|  | Gly | A: 144 | 2.34 | Conventional Hydrogen Bond |
|  | Glu | A: 183 | 5.99 | Carbon Hydrogen Bond |
|  | Asn | A: 101 | 3.03 | Carbon Hydrogen Bond |
|  | Glu | A: 183 | 3.56 | Pi-Anion |
|  | Ile | A: 171 | 5.37 | Alkyl |
|  | Pro | A: 173 | 4.45 | Alkyl |
|  | Lys | B: 254 | 5.46 | Pi-Alkyl |
| **Ligand Pred10 –** **Tubulin** | Val | B: 315 | 2.37 | Conventional Hydrogen Bond |
|  | Lys | B: 352 | 2.65 | Conventional Hydrogen Bond |
|  | Asn | A: 101 | 2.10 | Conventional Hydrogen Bond |
|  | Cys | B: 241 | 3.47 | Pi-Donor Hydrogen Bond |
|  | Lys | B: 254 | 3.46 | Halogen |
|  | Leu | B: 255 | 3.31 | Pi-Sigma |
|  | Ala | B: 250 | 4.86 | Pi-Alkyl |
|  | Lys | B: 254 | 4.72 | Pi-Alkyl |
|  | Leu | B: 248 | 5.33 | Pi-Alkyl |
|  | Lys | B: 254 | 4.81 | Alkyl |
| **Ligand Pred11 –** **Tubulin** | Asn | A: 101 | 2.29 | Conventional Hydrogen Bond |
|  | Asn | B: 258 | 2.23 | Conventional Hydrogen Bond |
|  | Cys | B: 241 | 3.63 | Pi-Donor Hydrogen Bond |
|  | Met | B: 259 | 4.95 | Pi-Sulfur |
|  | Leu | B: 255 | 3.30 | Pi-Sigma |
|  | Lys | B: 352 | 4.23 | Pi-Alkyl |
|  | Ala | B: 316 | 5.14 | Pi-Alkyl |
|  | Ala | B: 250 | 4.44 | Pi-Alkyl |
|  | Leu | B: 255 | 4.10 | Alkyl |
|  | Leu | B: 252 | 5.24 | Alkyl |
|  | Leu | B: 242 | 4.23 | Alkyl |
| **Ligand Pred12 –** **Tubulin** | Gly | A: 146 | 1.85 | Conventional Hydrogen Bond |
|  | Asn | A: 101 | 2.96 | Conventional Hydrogen Bond |
|  | Thr | A: 145 | 2.48 | Conventional Hydrogen Bond |
|  | Gln | A: 11 | 2.40 | Conventional Hydrogen Bond |
|  | Ala | A: 12 | 2.77 | Conventional Hydrogen Bond |
|  | Gln | A: 11 | 3.51 | Carbon Hydrogen Bond |
|  | Ala | A: 12 | 3.88 | Pi-Sigma |
|  | Tyr | A: 224 | 4.84 | Pi-Alkyl |
|  | Leu | B: 248 | 4.20 | Alkyl |
|  | Ala | A: 12 | 3.70 | Alkyl |
|  | Ile | A: 171 | 4.49 | Alkyl |
| **Ligand Pred13 –** **Tubulin** | Gly | A: 144 | 2.68 | Conventional Hydrogen Bond |
|  | Asn | A: 101 | 2.95 | Conventional Hydrogen Bond |
|  | Asp | A: 69 | 2.46 | Conventional Hydrogen Bond |
|  | Gly | A: 142 | 2.80 | Conventional Hydrogen Bond |
|  | Lys | B: 254 | 2.01 | Conventional Hydrogen Bond |
|  | Thr | A: 145 | 2.35 | Conventional Hydrogen Bond |
|  | Lys | B: 254 | 3.61 | Carbon Hydrogen Bond |
|  | Gln | A: 11 | 3.54 | Carbon Hydrogen Bond |
|  | Glu | A: 183 | 4.70 | Halogen |
|  | Tyr | A: 224 | 4.53 | Pi-Alkyl |
|  | Lys | B: 254 | 5.43 | Pi-Alkyl |
|  | Leu | B: 248 | 4.20 | Alkyl |
|  | Ala | A: 12 | 3.75 | Alkyl |
|  | Ile | A: 171 | 5.29 | Alkyl |
|  | Pro | A: 173 | 4.69 | Alkyl |
| **Ligand Pred14 –** **Tubulin** | Gly | A: 146 | 1.93 | Conventional Hydrogen Bond |
|  | Gly | A: 144 | 2.58 | Conventional Hydrogen Bond |
|  | Thr | A: 145 | 2.34 | Conventional Hydrogen Bond |
|  | Gly | A: 144 | 2.41 | Conventional Hydrogen Bond |
|  | Gly | A: 143 | 3.46 | Carbon Hydrogen Bond |
|  | Gln | A: 11 | 3.68 | Carbon Hydrogen Bond |
|  | Ala | A: 12 | 3.60 | Pi-Sigma |
|  | Tyr | A: 224 | 4.97 | Pi-Pi Stacked |
|  | Leu | B: 248 | 4.76 | Alkyl |
|  | Ala | A: 12 | 3.48 | Alkyl |
|  | Ile | A: 171 | 3.84 | Alkyl |
|  | Pro | A: 173 | 5.44 | Alkyl |
|  | Lys | B: 254 | 4.21 | Alkyl |
| **Ligand Pred15 –** **Tubulin** | Gly | A: 146 | 2.13 | Conventional Hydrogen Bond |
|  | Gly | A: 144 | 2.09 | Conventional Hydrogen Bond |
|  | Thr | A: 145 | 2.37 | Conventional Hydrogen Bond |
|  | Gly | A: 144 | 2.64 | Conventional Hydrogen Bond |
|  | Gly | A: 10 | 3.38 | Carbon Hydrogen Bond |
|  | Glu | A: 183 | 6.09 | Carbon Hydrogen Bond |
|  | Asn | A: 101 | 3.20 | Pi-Donor Hydrogen Bond |
|  | Leu | B: 248 | 4.20 | Alkyl |
| **Ligand Pred27 –** **Tubulin** | Val | B: 315 | 2.60 | Conventional Hydrogen Bond |
|  | Cys | B: 241 | 3.46 | Pi-Donor Hydrogen Bond |
|  | Leu | B: 255 | 3.31 | Pi-Sigma |
|  | Leu | B: 248 | 4.96 | Pi-Alkyl |
|  | Lys | B: 254 | 5.03 | Pi-Alkyl |
|  | Ala | B: 250 | 4.88 | Pi-Alkyl |
|  | Lys | B: 254 | 4.38 | Alkyl |
| **Ligand Pred28 –** **Tubulin** | Gly | A: 146 | 1.94 | Conventional Hydrogen Bond |
|  | Gly | A: 142 | 2.96 | Conventional Hydrogen Bond |
|  | Thr | A: 145 | 2.46 | Conventional Hydrogen Bond |
|  | Gln | A: 11 | 2.32 | Conventional Hydrogen Bond |
|  | Asn | A: 101 | 2.89 | Conventional Hydrogen Bond |
|  | Lys | B: 254 | 3.36 | Carbon Hydrogen Bond |
|  | Gln | A: 11 | 3.57 | Carbon Hydrogen Bond |
|  | Glu | A: 183 | 3.25 | Halogen |
|  | Gly | A: 142 | 3.16 | Halogen |
|  | Lys | B: 254 | 5.38 | Pi-Alkyl |
|  | Ala | A: 12 | 4.77 | Pi-Alkyl |
|  | Ile | A: 171 | 5.43 | Alkyl |
|  | Pro | A: 173 | 4.84 | Alkyl |
|  | Leu | B: 248 | 4.19 | Alkyl |
